# Glucose-Restricted Diet Regulates the Tumor Immune Microenvironment and Prevents Tumor Growth in Lung Adenocarcinoma

**DOI:** 10.3389/fonc.2022.873293

**Published:** 2022-04-29

**Authors:** Alexander Gähler, Denis I. Trufa, Mircea T. Chiriac, Patrick Tausche, Katja Hohenberger, Ann-Kathrin Brunst, Manfred Rauh, Carol I. Geppert, Ralf J. Rieker, Susanne Krammer, Anna Leberle, Markus F. Neurath, Horia Sirbu, Arndt Hartmann, Susetta Finotto

**Affiliations:** ^1^ Department of Molecular Pneumology, Friedrich-Alexander-Universität (FAU) Erlangen-Nürnberg, Universitätsklinikum Erlangen, Erlangen, Germany; ^2^ Department of Thoracic Surgery, Friedrich-Alexander-Universität (FAU) Erlangen-Nürnberg, Universitätsklinikum Erlangen, Erlangen, Germany; ^3^ Department of Medicine 1 - Gastroenterology, Pneumology and Endocrinology, Friedrich-Alexander-Universität (FAU) Erlangen-Nürnberg, Universitätsklinikum Erlangen, Erlangen, Germany; ^4^ Department of Paediatrics and Adolescent Medicine, Friedrich-Alexander-Universität (FAU) Erlangen-Nürnberg, Universitätsklinikum Erlangen, Erlangen, Germany; ^5^ Institute of Pathology, Friedrich-Alexander-Universität (FAU) Erlangen-Nürnberg, Universitätsklinikum Erlangen, Erlangen, Germany; ^6^ Comprehensive Cancer Center Erlangen-EMN (CCC ER-EMN), Erlangen, Germany

**Keywords:** NSCLC, Immunotherapy, glucose, BMI, IGFR, A549

## Abstract

**Background:**

Lung cancer is the second common cancer type in western countries and has a high mortality. During the development and progression of the tumor, the nutrients in its environment play a central role. The tumor cells depend crucially on glucose metabolism and uptake. Tumor cell metabolism is dominated by the Warburg effect, where tumor cells produce large amounts of lactate from pyruvate under aerobic conditions. We thus reasoned that, reducing carbohydrates in the diet might support anti-tumoral effects of current immunotherapy and additionally target tumor immune escape.

**Objectives:**

The link between reducing carbohydrates to improve current immunotherapy is not clear. We thus aimed at analyzing the effects of different glucose levels on the tumor development, progression and the anti-tumoral immune response.

**Methods:**

We correlated the clinical parameters of our LUAD cohort with different metabolic markers. Additionally, we performed cell culture experiments with A549 tumor cell line under different glucose levels. Lastly, we investigated the effect of low and high carbohydrate diet in an experimental murine model of lung cancer on the tumor progression and different immune subsets.

**Results:**

Here we found a positive correlation between the body mass index (BMI), blood glucose levels, reduced overall survival (OS) and the expression of Insulin-like growth factor-1 receptor (IGF1R) in the lung tumoral region of patients with lung adenocarcinoma (LUAD). Furthermore, increasing extracellular glucose induced IGF1R expression in A549 LUAD cells. Functional studies in a murine model of LUAD demonstrated that, glucose restricted diet resulted in decreased tumor load *in vivo*. This finding was associated with increased presence of lung infiltrating cytotoxic CD8+ T effector memory (TEM), tissue resident memory T (TRM) and natural killer cells as well as reduced IGFR mRNA expression, suggesting that glucose restriction regulates lung immunity in the tumor microenvironment.

**Conclusions:**

These results indicate that, glucose restricted diet improves lung immune responses of the host and suppresses tumor growth in experimental lung adenocarcinoma. As glucose levels in LUAD patients were negatively correlated to postoperative survival rates, glucose-restricted diet emerges as therapeutic avenue for patients with LUAD.

## Introduction

Lung cancer is the second common cancer type worldwide, with 12.4% of all cancer diagnoses and the highest lethality of all cancer-related deaths in the industrialized world (1, 2). Non-small cell lung cancer (NSCLC) accounts for approximately 85% of all lung cancer-related cases (3–5). Even though there are improving therapeutic strategies for lung cancer treatment, such as classic surgery, chemotherapy, radiation and immune therapy the 5-year survival rate remains poor with a median of 14% (6). In addition, the strong side effects of current anti-tumor therapies and the development of escape mutants represent a fundamental challenge.

The growth of tumor cells is crucially influenced by the nutrients in the surrounding of the tumoral area. One of the key nutrients for the survival and proliferation of the tumor is glucose. In healthy cells, glucose is taken up *via* glucose transporters (GLUT1-14) and is metabolized into pyruvate by glycolysis. Subsequently, the pyruvate can be used for fueling the tricarboxylic acid cycle (TCA cycle) and providing NADH for the mitochondrial ATP synthesis. In contrast, tumor cells mainly depend on aerobic glycolysis rather than oxidative phosphorylation. They produce lactate by fermentation also under aerobic conditions which is known as Warburg effect (7–9). This results in a reduced function of the TCA cycle and impaired mitochondrial function. The lactate that is released by the tumor cells leads to even more tumor proliferation and the inhibition of anti-tumoral activity.

Other possible mechanisms supporting tumor cell development are changes in systemic levels of growth hormones, such as somatotropic hormone (STH) and insulin-like growth factor 1 (IGF1). IGF1 plays a major role in tissue development and is an essential factor for bone and cartilage growth in childhood (10). IGF1 is produced by the liver and gets stimulated by somatotropin (STH). STH secretion is induced by Growth hormone releasing hormone (GHRH) and growth hormone (GH), secreted from the hypothalamus, induced by circulating glucose, fatty acids and amino acids (11). The expression of IGF1-receptor (IGF1R), a tyrosine kinase receptor, is upregulated in most cancer types, including prostate, breast and lung cancer (12). The IGF1R/IGF1 axis is a crucial progression factor for tumor cell development, supporting tumor cell survival, cell cycle, proliferation as well as cell growth, migration, invasion and angiogenesis (13, 14).

Furthermore, activation of IGF1R leads to changes in cell metabolism. Glycolysis and glucose uptake are mediated by the translocation into the cell membrane of Insulin-Responsive Glucose Transporter type 4 (GLUT-4). GLUT-4 translocation into the cell membrane is stimulated by Protein Kinase B (PKB, AKT) and plays a key role in glucose metabolism, apoptosis, cell proliferation, transcription and cell migration (15). The mechanistic target of rapamycin (mTOR) also depends on IGF1R signaling in cancer cells and directly leads to protein biosynthesis and anabolic metabolism.

In addition, previous studies have demonstrated that high extracellular glucose concentration results in epithelial to mesenchymal transition (EMT) in colorectal carcinoma (16) and uterus endometrial cancer cells (17). EMT is the morphologic transition of epithelial cells into mesenchymal like cells that gain the ability to migrate and therefore form metastasis. EMT is also associated with invasion in new tissues, supported by the upregulation of Snail Family Transcriptional Repressor 1 (SNAI1) (18) and Twist Basic Helix-Loop-Helix Transcription Factor 1 (TWIST1) (19) *via* Snail Family Transcriptional Repressor 2 (SLUG; SNAI2) regulation (20) in different cancer types (21–26).

Here we investigated the role of high extracellular glucose concentration in the lung tumor microenvironment (TME) and its role in controlling tumor growth *in vivo*.

## Material and Methods

### Human Subjects and Study Population

This study was performed at the Friedrich-Alexander-University of Erlangen-Nürnberg in Germany and was approved by the ethics review board of the University of Erlangen (Re-No: 56_12B; DRKS-ID: DRKS00005376). One hundredfortynine (149) patients suffering from Non-Small Cell Lung Cancer (NSCLC) underwent surgery and gave their approval to be enrolled in this study. Patients’ confidentiality was maintained.

Tissue samples were taken during surgery from the tumoral area (TU: solid tumor tissue), the peritumoral area (PT: 2 cm around tumor), as well as the tumor free control area (CTR: at least 5 cm away from the solid tumor) of the surgically removed lung tissue. A pathologist confirmed histologically the absence of tumor cells in the control region (CTR) and the presence of tumor in the Tumor region. We cannot exclude the presence of tumor cells in the PT region which we think is a place where tumor cells interact with the immune system.

Lung cancer diagnosis was confirmed immediately after lung surgery by the Institute of Pathology at the University Hospital Erlangen on histologic lung sections. Tissue samples were classified for histological subtype, according to the 2015 World Health Organization Classification of Lung Tumors. TNM Staging is based on the proposals for Revision of the TNM Stage Groupings in the Forthcoming (Eighth) (TNM8) Edition of the TNM Classification for Lung Cancer by the International Association for the Study of Lung Cancer (IASLC), issued in 2016. Relevant data for this study were provided by the Department of Thoracic Surgery and the Institute of Pathology, University Hospital in Erlangen and are listed in **Supplementary Tables S1–S3**.

### RNA Extraction and Quantitative Real-Time PCR

Total RNA was extracted from frozen tissue samples or from cell suspension samples, using Qiazol Lysis^®^ Reagent (QIAGEN, Cat#79306) according to the manufacturer’s instructions. 1 µg of the resulting RNA was reverse transcribed into copy DNA (cDNA) *via* the RevertAid™ First Strand cDNA Synthesis Kit (ThermoFisher Scientific, Cat#K1622) according to the manufacturer’s protocol. Each qPCR reaction mix contained 15 ng of cDNA, 300 nM transcript-specific forward and reverse primer iTaq Universal SYBR Green Supermix (Bio-Rad Laboratories, Cat# 1725124) in a total volume of 20 µl. qPCR primers were purchased from Eurofins Genomics Germany GmbH (Ebersberg, Germany). Primer sequences for murine and human qPCR analysis are shown in the **Supplementary Table S4**. Reactions were performed for 50 cycles with an initial activation for 2 min at 98°C, denaturation for 5 min at 95°C and hybridization and elongation for 10 min at 60°C. qPCR reactions were performed using the CFX-96 Real-Time PCR Detection System and analyzed *via* the CFX Manager Software (both Bio-Rad Laboratories GmbH, Feldkirchen, Germany). The relative expression level of specific transcripts was calculated using the relative quantification 2-ΔΔCT method with respect to the internal standard glycerinaldehyd-3-phosphat-dehydrogenase (GAPDH) and data with ΔCq values higher than 35 were excluded.

### A549 Cell Culture Experiment

The human A549 (ATCC^®^ CCL-185™) cell line was purchased authenticated from the ATCC bank (Manassas, Virginia, USA). 1x10^5^ A549 cells were cultured in 2 ml medium for 72 h in 6 well plates (Greiner Bio-One, Cat#657160). The applied RPMI contained 0 mg/dl glucose, RPMI 100 mg/dl glucose or RPMI 200 mg/dl glucose. Cells were either left unstimulated or were treated with 100 ng/ml of rhIGF1 (R&D Systems, Cat#291-G1). Cells were incubated at 37°C, 5% CO2 and 96% relative humidity. After 72h, cells and supernatants were collected for further analysis. Used media are listed in **Supplementary Table S5**.

### Proliferation Assay of A549 Cells

Analysis of proliferating A549 cells was performed using Ki-67 staining. A549 cells were collected, centrifuged and supernatant was removed. To each cell pellet, 500 µl of ice-cold 75% ethanol was added dropwise while vortexing and fixed cells were stored for at least 48 hours at –20°C. Fixed cells were then transferred in a 96-well plate (U-bottom, Greiner Bio-One, Cat# 650161), washed twice with 200 µl wash buffer (PBS (anprotec, Cat# AC-BS-0002), 1% FCS) and resuspended in 100 µl wash buffer. For Ki-67 staining 20 µl of FITC mouse anti-Ki-67 antibody (FITC Mouse Anti-Ki-67 Set, BD Biosciences, Cat# 556026) were added to each well, mixed gently and incubated at room temperature for 30 min in the dark. The reaction was stopped by adding 200 µl of wash buffer, followed by centrifugation (300 rpm, 10 min, 4°C) and resuspension in 100 µl wash buffer. Cells were then analyzed by flow cytometry (FACS Canto II, BD Biosciences) and data sets were evaluated by Flow-Jo v10.2.

### Apoptosis Assay of A549 Cells

Analysis of apoptotic A549 cells was performed using Annexin V/PI staining according to the manufacturer’s protocol (BD Bioscience; Cat#550474 and #556463). Briefly, 1x10^5^ cells were stained with 5 μl AnnexinV-APC and 5 μl PI in 100 μl 1x AnnexinV binding buffer for a total volume of 110 μl in 5 ml FACS tubes and incubated for 15 min at RT in the dark. Reaction was stopped by adding 200 μl 1x AnnexinV binding buffer and samples were immediately analyzed with the FACSCanto II. Data sets were evaluated by Flow-Jo v10.2.

### Wound Healing Assay

For the analysis of A549 cell migration, 70µl with 5x10^4^ cells per ml were seeded into a cell culture insert (culture-Insert 2 Well for self-insertion, ibidi, Cat# 80209) in a 24-well cell culture plate (Greiner Bio-One, Cat# 662160). Cells have been cultured to adhere for 24h in RPMI 100 mg/dl glucose and were grown to 90% confluence. Cells were incubated at 37°C, 5% CO2 and 96% RH. By taking out the culture insert, a simulated wound was created with 500µm distance. Cells were washed with 1 ml PBS. New RPMI 100 mg/dl glucose was added and left either unstimulated or treated with 100 ng/ml of IGF1. Cell migration were detected over a period of 48 h using JuLI™ Br live cell movie analyzer (NanoEnTek). The extent of healing was defined by the ratio of the difference between the resulting wound areas compared with the original wound area. Therefore, QuPath v0.2.3 was used. Used media are listed in **Supplementary Table S5**. Antibodies that were used are listed in **Table S6**.

### Glucose and Lactate Detection

To analyze the glucose and lactate concentration in cell culture supernatants, a Cobas 6000 (Roche) analyzer was used. Measurements were performed in collaboration with the laboratory of the children’ clinic Erlangen, according to the manufacturer’s instructions.

### Flow Cytometric Analysis (FACS)

For flow cytometric analysis of surface antigen expression, single-cell suspensions of 0.2-2x10^6^ cells were transferred into U-bottom 96 well plates (Greiner Bio-One, Cat# 650161) and centrifuged. All centrifugation steps were performed at 1 min, 2000 rpm and 4°C (Thermo Fisher Scientific, Heraeus Megafuge™ 16R, Cat# 75004270). The supernatant was discarded, and the cell pellets were incubated with unlabeled monoclonal anti-CD16/CD32 antibodies diluted 1:100 in FACS buffer (PBS; 2% FCS) for at least 5 min at 4°C in the dark to block non-specific binding of antibodies to Fc receptors. Cells were washed with 150 µl FACS-buffer (PBS + 2% FCS), centrifuged and the supernatant was removed. Next, cell pellets were resuspended in 50 µl FACS buffer supplemented with fluorochrome-coupled antibodies in appropriate concentrations and incubated for 15 min at 4°C in the dark. Cells were washed with 150 µl FACS buffer, centrifuged and resuspended in 200 µl FACS buffer to be analyzed immediately with the FACS Canto II (BD Biosciences, Heidelberg). The stained cell pellets were resuspended in 100µl FACS-buffer and measured with FACS Canto II (BD Biosciences, Heidelberg). The antibodies used in this study are listed in **Supplementary Tables S6–S8**. Data sets were analyzed with softwares Flow-Jo v10.2 (FlowJo, LLC, Oregon, USA) or Kaluza (Beckman Coulter, Krefeld, Germany).

### Mouse Lung Cancer Model

All mice were bred and maintained under specific-pathogen-free (SPF) conditions at our animal facility (University Hospital Erlangen, Hartmannstraße 14, Erlangen). All experiments were performed in accordance with the German and European laws for animal protection and were approved by the local ethics committees of the Regierung Unterfranken (Az 55.2-2532-2-1286-20).

Eight weeks old mice (C57BL/6J mice-female) were purchased from Janvier Labs and two days before tumor cell injection, the food was changed in accordance to the experimental protocol described in **Figure 5A**. The mice were fed with carbohydrate free food (Altromin, C-1009), high carbohydrate food (Altromin, C-1000) and breeding food (Altromin, C-1324) for 24 days. The composition and micro nutrition of the food is shown in **Supplementary Table S10**. During the diet, all mice were sat under infrared (IR) light for 6 hours daily in a distance of one meter to support body temperature.

For the induction of non-small cell lung cancer, mice were injected intravenously with 5 x10^5^ LL/2-luc-M38 cells (Caliper LifeScience) in 200 μl DMEM Medium without additions in the tail vein. LL/2-luc-M38 is a luciferase expressing cell line that was generated from murine lung carcinoma LL/2 cells by stable transfection of the North American Firefly Luciferase gene expressed from the PCI-luc promoter. For quantification of the tumor load *in vivo*, bioluminescence imaging has been performed on day 16 and day 20, respectively. Therefore, luciferin (0.15 mg/g body weight; Promega, Cat#P1043) was injected intraperitoneally. Fifteen minutes after injection, mice were sedated with Isoflurane for 5 min and placed in the *in vivo* imaging system (IVIS). The intensity of luminescence represents the load of the tumor and the average radiance (p/s/cm2/sr) of an area 3,17 cm x 3,17 cm was applied for analysis.

### Lung Cell Isolation and Culture

Lung single cell suspensions were prepared by cutting the resected lung into small pieces, followed by collagenase treatment (Collagenase from Clostridium histolyticum; Cat# C98991-500MG, 2700 U/ml; Sigma-Aldrich) and 1,5 mg DNase (DNase I, 10 mg/ml; Cat#10104159001, Roche Diagnostics GmbH) diluted in 10 ml RPMI medium containing 0 mg/dl or 200 mg/dl glucose in a shaker (Edmund Bühler GmbH, Inkubationshaube TH 15) for 45 minutes at 37°C. Cells from mice fed with low carbohydrate diet were diluted in RPMI with 0 mg/dl glucose. Cells from mice fed with breeding diet and high carbohydrate diet were diluted in RPMI with 200 mg/dl glucose. The lung digest was then filtered through a 40 µm cell strainer (Greiner Bio-One, Cat#542040). Afterwards the cells were centrifuged (10 min, 1500 rpm, 4°C). Supernatant was removed and cells were resuspended with ACK-lysis buffer (0,15M NH4Cl, Carl Roth GmbH + Co. KG, Cat# P726.2; 0,01M KHCO3, Carl Roth GmbH + Co. KG, Cat#P748.1; 100M Na2EDTA, GERBU Biotechnik GmbH, Cat# 1034.1000; dissolved and sterile filtered in deionized H2O; pH=7.2-7.4) and incubated for 2 min, followed by centrifugation as described above. Supernatant was removed and the cells were resuspended in PBS (Anprotec, Cat# AC-BS-0002, Bruckberg, Germany). Next, the suspension was transferred in a 10 ml pipette and slowly filled into the falcon to remove fat. The suspension was then centrifuged (5 min, 1500 rpm, 4°C) and resuspended in 10 ml PBS. Cell number was determined by using Trypan-blue staining in a Neubauer counting chamber.

For *in vitro* lung cell culture, 1 x 10^6^ cells of total lung cell suspension were seeded in 48 well plate with RPMI medium containing 0 mg/dl or 200 mg/dl glucose as described above. Cells were either left unstimulated or stimulated with 500 ng/ml of plate-bound anti-CD3 and 2000 ng/ml anti-CD28 antibodies. Cells were cultured for five days in an incubator at 37°C and 5% CO2. Supernatant was then collected and used for ELISA or cytotoxic assay. Cells were harvested with Qiazol Lysis^®^ Reagent (QIAGEN, Maryland, USA, Cat# 79306) according to the manufacturer’s instructions. Media are listed in **Supplementary Table S5**. Recombinant proteins used for cell culture are listed in **Supplementary Table S7**.

### Hematoxylin and Eosin Staining on Murine Paraffin-Embedded Lung Sections

Lung lobes were removed, fixed in 10% formalin-PBS solution, dehydrated, and embedded in paraffin. Five-micrometer-thick lung sections from paraffin blocks were stained with hematoxylin and eosin for visualization of lung tumors. Stained slides were then scanned using a digital slide scanner (Scan 150, 3D Histech Ltd). Slide images were analyzed with the CaseViewer software (Version 2.0, 3D Histech Ltd). Hematoxylin and eosin staining were performed in collaboration with the Institute of Pathology at the University Hospital Erlangen.

### Histologic Tumor Load Detection

Tumor load was identified by QuPath v0.2.3. Tumor load was defined as the ratio between total lung tissue and tumor shown in histology on one slide. Total lung tissue was identified with simple tissue detection tool and distinct bronchiole areas were substractes. For verification, the lung was analyzed histologically by a pathologist from the Institute of Pathology at the University Hospital Erlangen in a blinded study.

### Bioluminescence Cytotoxic Assay

For cytotoxic analysis of total lung cell supernatant, we used LL/2-luc-M38 cells as a target. 7×10^3^ cells per well were seeded in 96-well white-walled plates (Berthold, Cat#23300) in quadruplicates. LL/2-luc-M38 cells were cultured in DMEM medium, which was also added as a negative control without containing cells. The cells were cultured for 24h at 37°C in 5% CO_2_. This reporter assay uses the bioluminescence reaction of the enzyme luciferase and its substrate luciferin. In our case the bioluminescence signal is proportional to the luciferase expressing LL/2-luc-M38 cells maintained in their function after the cytotoxic challenge. After 24h, the supernatant was discarded and cells were washed with 100 μl PBS. To evaluate cytotoxic activity of lung cell culture supernantants, LL/2-luc-M38 received the respective medium or supernatant as challenge. Therefore, LL/2-luc-M38 were treated with supernatants of the five days cell culture of murine lung cells diluted 1:3 with RPMI 200 mg/dl glucose or RPMI 0 mg/dl glucose, respectively (as indicated in the results section). As a positive control we treated the LL/2-luc-M38 cells with supernatant of the cytotoxic CD8+ T cell line CTLL2 which was kindly provided by Dr. Ulrike Schleicher (Institute of Microbiology in Erlangen). The supernatant was gained from 1.2x10^6^ CTTL2 cells cultured in 30ml RPMI1640 medium supplemented 4 ng/mL mIL-2 (ImmunoTools Cat#12340024) at 37°C and 5% CO2. After change of media the cells were cultured for another 24h. At 48h of culture, the medium of all wells was removed and wells were carefully washed with 37°C warm PBS to remove residual culture media. Afterwards, 100 μl of luciferin (150 μg/ml diluted in PBS; Promega, Cat#P1043) was added and cells were incubated for 25 min at 37°C in 5% CO2. Bioluminescence measurements were performed with 0.5 s counting time at room temperature using a Centro XS3 LB 960 plate-reading luminometer (Berthold Technologies, Bad Wildbad, Germany). For Analysis, the mean of quadruplicates was calculated. Respective cell numbers surviving after the cytotoxic challenge were calculated by using the standard curve obtained from quadruplicated of increasing known numbers of LL/2-Luc-M38 cells. Media are listed in **Supplementary Table S5**.

### Enzyme-Linked Immunosorbent Assay

The enzyme-linked immunosorbent assay (ELISA) technique was utilized to analyze the cytokine concentration in cell culture supernatant and was performed according to the manufacturer’s instructions. Murine ELISA for IFN-γ (Cat#555138), IL-2 (Cat#555148), IL-10 (Cat#555252) were purchased from BD Bioscience, and murine ELISA for TGF-β (Cat#DY9679) was purchased from R&D Systems. ELISA kits are listed in **Supplementary Table S9**.

### Statistical Analysis

Differences were evaluated by using GraphPad Prism 8 software to obtain significance levels (*P,0.05; **P,0.01; ***P,0.001; ****P,0.0001). Data were imported in column statistics and were then analyzed for normal distribution with Shapiro-Wilk test and Kolmogorov-Smirnov test. For normal distribution, one-way ANOVA multiple comparisons was used. For non-normal distribution or n<5 the Kruskal-Wallis test was used. For two independent groups and normal distribution unpaired t-test was used. For non-normal distribution, Mann-Whitney test was chosen. Correlations were examined by linear regression curve. The two-tailed Pearson correlation analysis was performed to get the r and p value. P-values were adjusted and *post hoc* analysis were performed using Bonferroni-Holm correction. Survival data are represented in Kaplan–Meier survival curves. Log rank test was used to compute p values.


Data are given as mean values ±SEM

## Results

### BMI, Blood Glucose Levels and IGF1R mRNA Expression Level Correlated With the Overall Survival of Patients With Lung Adenocarcinoma

**Figure 1:**
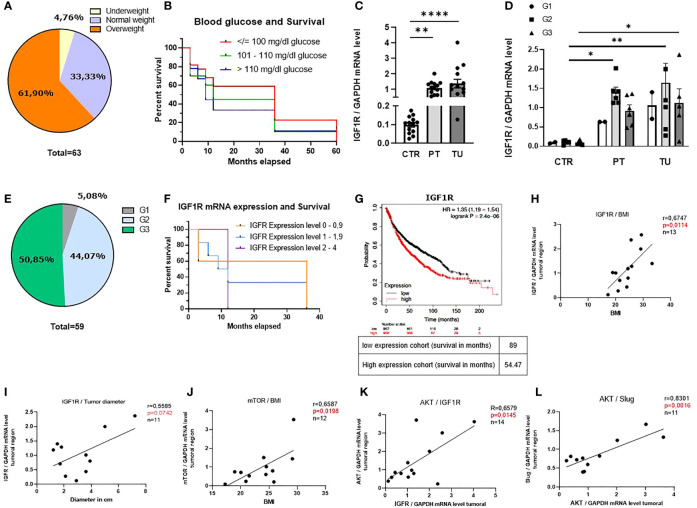
BMI, blood glucose levels and IGF1R mRNA expression level influence the overall survival of patients with lung adenocarcinoma (LUAD). **(A)** Diagram of distribution of our patients cohort depending on the BMI (underweight: n=3, normal weight: n=21, overweight: n=39). **(B)** Kaplan-Meier curve depending on patients’ blood glucose level the day before surgery; (blood glucose </= 100 mg/dl n=22, blood glucose 101 - 110 mg/dl n=20 blood glucose >110 mg/dl n=9). **(C)** qPCR analysis of relative IGF1R/GAPDH mRNA expression level in patients with LUAD: control region (CTR), peritumoral region (PT) and tumoral region (TU). (CTR n=13; PT n=14 TU n=14, p (CTR vs. PT)=0,001 and p (PT vs. TU)=<0,0001). **(D)** qPCR analysis of relative IGF1R/GAPDH mRNA expression level differentiated for tumor grading of patients with LUAD. Data are shown as mean values ± s.e.m. (n=15); p<0,05. **(E)** Diagram of distribution of our patients cohort depending on the grading (G1:n=3, G2:n=26, G3:n=30). **(F)** Kaplan-Meier curve depending on patients IGF1R mRNA expression level; (0 – 0,9 n=5, 1 - 1,9 n=6, 2 – 4 n=3). **(G)** Cancer Genome Atlas Program (TCGA) and KMplot data analysis of IGF1R mRNA; p= 0.0059, n= 2437. **(H)** qPCR analysis of relative IGF1R/GAPDH mRNA expression level correlated with BMI of patients (n=13). p= 0,0114, r=0,6747 (B-H p= 0,0125). **(I)** qPCR analysis of relative IGF1R/GAPDH mRNA expression level correlated with tumor diameter (in cm) of patients (n=11)), p= 0,5585, r=0,07042, (B-H p= 0,05). **(J)** qPCR analysis of relative mTOR/GAPDH mRNA expression level correlated with BMI of patients (n=12), p= 0,0198, r=0,6587, (B-H p= 0,025). **(K)** qPCR analysis of relative AKT/GAPDH mRNA expression level correlated with relative IGF1R/GAPDH mRNA expression level of patients (n=14); p= 0,0145, r=0,6579 (B-H p= 0,016). **(L)** qPCR analysis of relative Slug/GAPDH mRNA expression level correlated with relative AKT/GAPDH mRNA expression level of patients (n=11), p= 0,0016, r=0,8301 (B-H p= 0,0166). One-way ANOVA test was used for figure **(C, D)**. Correlations are shown using simple linear regression. The two-tailed Pearson correlation analysis was performed to get the r and p value for figure **(H–L)**. *Post hoc* analysis was computed using Bonferroni-Holm **(C, D)** correction and are referred to each correlation. Survival data are represented in Kaplan–Meier survival curves. Log‐rank test was used to compute P‐values for panel **(B, F, G)** Data are shown as mean values ± s.e.m.; *P,0.05; **P,0.01, ****P,0.0001.

To start investigating the connection between the nutrition status, overall survival (OS) and IGF1R expression level in patients with LUAD, we first differentiated our patient’s cohort into BMI intervals as follows: <18.5 (underweight), 18.5-24.9 (normal weight) and >24.9 (overweight) (27). The main characteristics of the patients analyzed in this study are reported in **Supplementary Tables S1–S3**. We used the BMI as an indicator for food intake and body fatness. We found that most patients were overweight, while only a minority of patients had underweight (**Figure 1A**). Interestingly, patients with lower BMI survived the most (**Figure S1A**). We next asked whether the blood glucose level, measured the day before surgery correlated with the overall survival and noticed that patients with glucose levels over 101 mg/dl had worse 3-year survival rates compared to patients with blood glucose level < 101 mg/dl (**Figure 1B**).

The insulin-like growth factor 1 receptor (IGF1R) is a transmembrane tyrosine kinase receptor that controls glucose metabolism (28). In order to investigate the role of IGF1R in the development of LUAD, we isolated mRNA samples obtained from control, peri-tumoral and tumoral regions of lung tissue from our cohort of patients with LUAD immediately after surgery (**Supplementary Figure S1B**) and performed a quantitative real time PCR (qPCR). Here, we found increased IGF1R mRNA expression level in the peri-tumoral and tumoral regions as compared to their control region of the lung of patients with LUAD (**Figure 1C**). We next considered the different gradings of lung cancer and discovered that patients with LUAD at stage G2 had higher levels of IGF1R in their peri-tumoral region of the lung compared to the control regions (**Figure 1D**). This group represents 44% of the patients analyzed (**Figure 1E**). To investigate whether IGF1R expression influences the survival of these patients, we correlated different ranges of mRNA expression levels (0-0.9, 1-1.9 and 2-4) with the overall survival and detected worse 3-year survival rates of patients with higher IGF1R mRNA expression levels as compared to patients with lower levels (**Figure 1F**). To assess the prognostic value of this potential biomarker we performed additional analyses of larger patient cohorts using the Cancer Genome Atlas Program (TCGA) and Kaplan-Meier plot analysis from a publicly accessible transcriptomic database (KMplotter) (29). This analysis showed that LUAD patients with reduced mRNA expression levels of IGF1R in the lung, have significantly better overall survival (OS) compared to patients with high IGF1R mRNA expression levels (**Figure 1G**), thereby confirming our findings on a larger cohort of patients. Next, we analyzed the downstream signaling of the IGF1/IGF1R axis in the tumoral region. Here, we noticed a correlation between IGF1R mRNA expression levels in the tumoral region and the BMI (**Figure 1H**) as well as with the tumor diameter, a marker for tumor development and growth (**Figure 1I**). In addition, a correlation between the BMI and the downstream IGF1R signaling molecule mTOR was detected (**Figure 1J**). Altogether, these data indicate that high BMI, high blood glucose levels and increased IGF1R expression correlate with reduced OS in patients with LUAD. In subsequent studies, we analyzed the downstream signaling molecules of IGF1R in the tumoral region of our cohort of LUAD patients. We observed a direct correlation between mRNA expression levels of IGF1R and the proliferation marker AKT (**Figure 1K**). Moreover, we found a direct correlation between AKT and *SLUG* mRNA expression levels (**Figure 1L**). A positive correlation between *SLUG* mRNA and BMI and IGF1R and *SLUG* mRNA expression levels (**Figures 1S C, D**) points out that IGF1R/IGF1 axis regulates *SLUG* and might be further involved in the formation of metastasis.

### High Glucose in the Lung Cancer Cell Microenvironment Activated Cell Cycle and Cell Metabolism and Upregulated the Expression of IGF1R in LUAD A549 Lung Adenocarcinoma Cell Line

**Figure 2:**
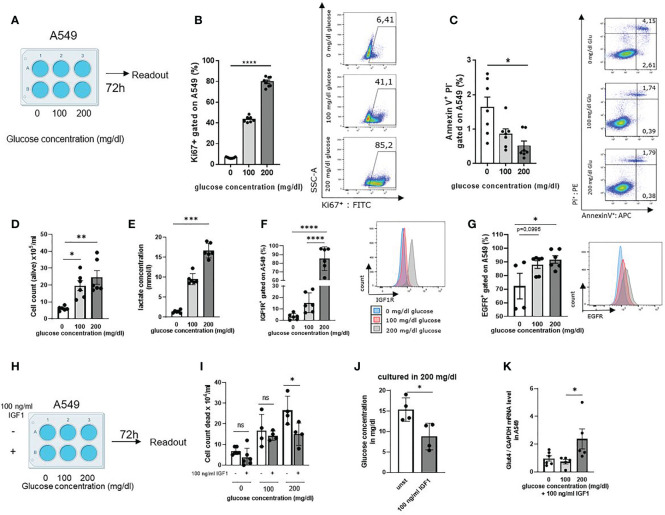
High glucose in the lung cancer cell microenvironment activated cell cycle and cell metabolism and upregulated the expression of IGF1R in LUAD A549 lung adenocarcinoma cells line. **(A)** Schematic illustration of experimental design. 5 x 10^5^ cells per well were cultured for 72 h in a 6 well plate. The extracellular glucose concentration was increased from 0 mg/dl over 100 mg/dl to 200 mg/dl. **(B)** Untreated A549 cells were stained with anti-Ki67 antibody and analyzed by flow cytometry; p (0 vs. 200 mg/dl glucose)=<0,0001 (n=7-8). **(C)** Annexin V/PI FACS analysis of untreated A549 cells culture, cultured as described in A; For early apoptosis, Annexin V+/P- p (0 vs. 200 mg/dl glucose)=0,0102 (n=7). **(D)** Cell count of alive A549 cells stained with trypan blue solution and cultured as described in a; p (0 vs. 100mg/dl glucose) = 0,0242 and p (0 vs. 200mg/dl glucose) =0,0043 (n=6). **(E)** Lactate concentration was measured in the supernatant after 72h cell culture. Cells were cultured as described in a; p (0 vs. 200 mg/dl glucose)=0,0003 n=6. **(F)** IGF1R expression on A549 cell surface after 72h cell culture, cultured as described in A and measured by flow cytometry; p (0 vs. 200 mg/dl glucose)=<0,0001, p (100 vs. 200 mg/dl glucose)=<0,0001, (n=6). **(G)** EGFR expression on A549 cell surface after 72h cell culture, cultured as described in A and measured by flow cytometry p (0 vs. 100 mg/dl glucose)=0,0995 p (0 vs. 200 mg/dl glucose)=0,0384, (n=4). **(H)** Schematic illustration of experimental design. Cells were cultured for 72 h in a 6 well plate 5 x 10^5^ cells per well, either unstimulated or stimulated with 100 ng/ml IGF1. The extracellular glucose concentration was increased from 0 mg/dl over 100 mg/dl to 200 mg/dl. **(I)** Cell count of dead A549 cells stained with trypan blue solution and cultured as described in **(H)**; p (200mg/dl glucose without IGF1 vs. 200mg/dl glucose with IGF1)=0,0328 (n=4). **(J)** Glucose concentration in the supernatant of cell culture with and without 100 ng/ml IGF1 cultured with 200 mg/dl glucose concentration after 72h; p (unst vs. 100 ng/ml IGF1)=0.0229 (n=4). **(K)** qPCR analysis of relative Glut4/GAPDH mRNA expression level in A549 cells cultured as described in **(H)**. with 100 ng/ml IGF1; p (100 vs. 200 mg/dl glucose)=0,0449 (n=5). One-way ANOVA test was used for figure **(B, C, F, G, I, K)** Kruskal-Wallis test was used for figure **(D, E)** Mann-Whitney test was used for figure **(J)** Data are shown as mean values ± s.e.m.; *P,0.05; **P,0.01, ***P, 0.001; ****P,0.0001 of at least three independent experiments.

In order to understand the effects of glucose in the cancer cell microenvironment, we next cultured the LUAD cell line A549 for 72h in RPMI 0 mg/dl, 100 mg/dl and 200 mg/dl glucose concentrations in the cell culture medium (**Figure 2A**, **Supplementary Table S5**). After 72h, cells were collected and stained with Ki-67, a proliferation associated marker that is detectable during DNA synthesis in S, G2 and M-phase of the cell cycle, but not in G1 and G0 (30). The gating strategy is shown in **Supplementary Figure S2**. Analyzing Ki-67 in the A549 cell cultures, we found increased Ki-67 levels upon increasing glucose concentrations (**Figure 2B**). Furthermore, to investigate if glucose would rescue cancer cell death by inhibiting programmed cell death, also known as apoptosis, we stained with Annexin-V and Propidium iodide (31). Here, we discovered that glucose rescued the cell death of A549 cells by inhibiting early and late apoptosis (**Figure 2C**, **Supplementary Figure 1E**). In agreement with these results, we noticed higher numbers of living cells by increasing glucose as compared to incubation settings with low glucose levels counting cells (**Figure 2D**). Next, we wanted to investigate if the tumor cell metabolism was also influenced by glucose. Therefore, the lactate concentration in the A549 cell culture supernatants was measured. Here we noticed significantly increased lactate concentrations in 200 mg/dl glucose cultures, indicative for increased aerobic glucose metabolism compared to cell cultures with lower glucose levels (**Figure 2E**). To understand whether glucose plays a role in IGF1R expression, we analyzed A549 cells after 72h cell culture by flow cytometry. Here we detected that A549 cancer cells cultured with higher extracellular glucose concentrations have upregulated IGF1R (**Figure 2F**) and EGFR expression on their cell membrane (**Figure 2G**). As these findings indicated that IGF1R expression depends on extracellular glucose concentration, we started to culture A549 cells under different glucose conditions with the IGFR1 ligand Insulin-like growth factor 1 (IGF1) at 100 ng/ml concentration (**Figure 2H**). In literature, IGF1 has been shown to bind to insulin receptors to stimulate glucose transport in fat and muscle (32, 33). Therefore, we wanted to see if IGF1 would also contribute to glucose uptake in the LUAD cell line A549. As the IGF1R/IGF1 axis is known to prevent tumor cell death, we counted the dead cells in the cell culture. Here we discovered a reduction of dead cells in those cell cultures treated with 100 ng/ml IGF1 and high glucose concentration compared to the cell culture without treatment (**Figure 2I**). Next, we wanted to investigate whether IGF1 influences glucose uptake into the tumor cells. IGF1 increased glucose uptake (**Figure 2J**) compared to the unstimulated cell culture medium when providing 200 mg/dl glucose. Next, we measured GLUT4 mRNA expression levels in A549 cell line as GLUT4 is important for insulin dependent glucose uptake. We found a significant induction of GLUT4 in IGF1 treated cells cultured with 200 mg/dl glucose as compared to 100 and 0 mg/dl glucose (**Figure 2K**).

Since we found high mRNA expression levels of IGF1R in tumor cells, we wanted to understand whether IGF1R/IGF1 axis plays a role in cell migration. Therefore, we started a series of cell scratch experiments with the human adenocarcinoma cell line A549. Tumor cells were grown in RPMI with 100 mg/dl glucose until a confluence of 80% was reached. The cells were then scratched and treated with 100 ng/ml IGF1 or left untreated. The scratch was monitored for 24h and analyzed for wound healing around the injury. Here we found that stimulation with 100 ng/ml IGF1 induces more rapid tumor cell migration as evaluated by healing of the scratched area (marked in red) (**Figures S1F, G**).

As we used the BMI as an overall indicator for food intake in our human cohort, we wanted to specify if *SLUG* is influenced by extracellular glucose concentration. Therefore, we next analyzed *SLUG* mRNA expression level in A549 cell line cultured with different extracellular glucose concentrations as already described above and either stimulation with 100 ng/ml hrIGF1 or left unstimulated. Here, we found a trend that increasing glucose leads to increasing *SLUG* mRNA expression levels. Moreover, stimulation with IGF1 induces *SLUG* expression significantly in addition to 200mg/dl glucose concentration in the cell culture medium (**Figure S1H**). These results suggest that IGF1 induces glucose consumption in the TME and promotes GLUT4 expression. Higher glucose levels and the IGF1R/IGF1 axis promote tumor development, inhibit tumor cell death and activate glucose metabolism in human lung adenocarcinoma cells.

### Carbohydrate Deprivation in the Diet Inhibited Lung Tumor Development in a Murine Model of Lung Cancer (LUAD) in a Prophylactic Setting and Increased OS Rate

**Figure 3:**
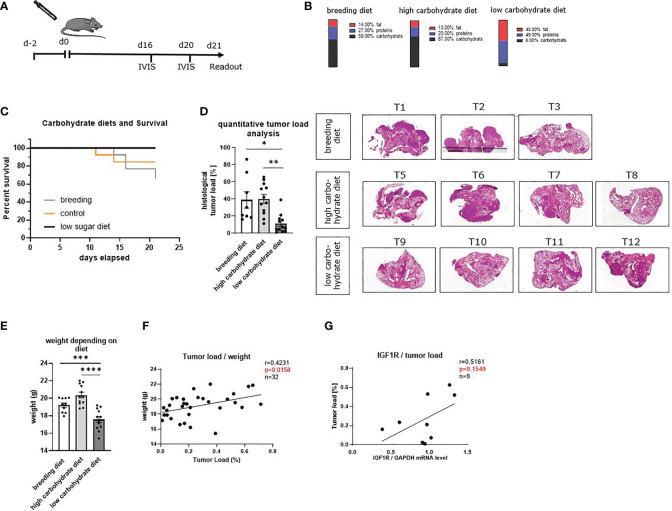
Sugar deprivation in the diet inhibited lung tumor development in a murine model of lung cancer (LUAD) in a prophylactic setting and increased OS rate. **(A)** Schematic illustration of a murine model of lung cancer. Two days before tumor cell injection, the food was changed to a high carbohydrate diet and low carbohydrate diet. One group still received the breeding diet. On day 0 tumor cells were injected in the mice’s tail vein. On day 16 and day 20 after tumor cell injection, *in vivo* bioluminescence imaging was performed. The readout was on day 21. **(B)** Diet composition of the different food used. The breeding food, usually used for our mice, a high carbohydrate diet with high amounts of carbohydrates (67%) and a low carbohydrate diet, containing 6% carbohydrates. **(C)** Kaplan- Maier curve, showing OS of mice fed with different diets. **(D)** Quantitative analysis of total tumor load shown in histology of three independent experiments, analyzed by QuPath; (n^breeding diet^=8, n^high carbohydrate diet^=12, n^low carbohydrate diet^=12), p (breeding diet vs. low carbohydrate diet) =0,0135, p (high carbohydrate vs. low carbohydrate diet) =0,0011. Representative histology of HE-stained lung slides from one lobe of the lung of tumor bearing mice. **(E)** weight of the mice depending on the diet; (n^breeding diet^=10, n^high carbohydrate diet^=12, n^low carbohydrate diet=^12), p (breeding diet vs. low carbohydrate diet) =<0,0031), p (high carbohydrate vs. low carbohydrate diet) =<0,0001) **(F)** direct correlation between the weight of the mice and the quantitative tumor load; n=31, r=04231, p=0,0158. **(G)** Direct correlation between the tumor load and IGF1R mRNA expression level in total lungs cells, n=9, r=0,5161, p=0,1549. One-way ANOVA test was used for panel **(D, G)** Correlations are shown using simple linear regression. The two-tailed Pearson correlation analysis was performed to get the r and p value for figure **(F, G)** Data are shown as mean values ± s.e.m.; *P,0.05; **P,0.01, ***P, 0.001; ****P, <0.0001 of at least three independent experiments.

To understand the effects of low glucose levels on LUAD development *in vivo*, we used an established murine model of lung cancer. Therefore, LUAD (LL/2) cells were injected intravenously, and mice were fed with a low carbohydrate diet, a high carbohydrate diet and a breeding diet (**Figure 3A**, **Supplementary Table S10**). The diets have different compositions. The standard breeding diet contained 59% carbohydrates, 27% proteins and 14% fat. In addition, we used a high carbohydrate diet, containing 67% carbohydrate, 20% protein and 13% fat, whereas the diet with decreased carbohydrates contained 6% carbohydrate, 49% protein and 45% fat (**Figure 3B**). In the *in vivo* experiments, we noticed that mice with a low carbohydrate diet have a higher survival rate compared to both other groups (**Figure 3C**). The diets were changed two days before injection of LUAD cells, and mice were fed consistently for 21 days. On day 21, after ending the experiment, the lungs of the mice were removed, and lung cells were dissociated, cultured and analyzed. To detect the development of the tumor load during the experiment, *in vivo* bioluminescence imaging was performed on day 16 and day 20 (**Supplementary Figures S3A–C**). For quantitative analysis of the *in vivo* bioluminescence measurement, we calculated the total tumor load as average radiance of the bioluminescence and found decreased tumor load in mice fed with the low carbohydrate diet compared to both other diets on day 16 and day 20. To verify the *in vivo* imaging finding showing a trend towards reduction of tumor load in the group fed with carbohydrate low diet (**Supplementary Figures S3A–C**), one lung lobe was analyzed histologically at the end of the experiment (day 21). Therefore, lung slides were stained with hematoxylin and eosin (HE) and analyzed in a blinded study by an expert pathologist or quantitatively with QuPath (**Figure 3D**, **Supplementary Figure 3D**, right handside). Consistent with the *in vivo* bioluminescence imaging, we found a decreased tumor load in the mice fed with low carbohydrate diet compared to the high carbohydrate diet group and the group fed with breeding diet. Furthermore, we analyzed the weight of the mice and found decreased weight in mice fed with the low carbohydrate diet (**Figure 3E**). Additionally, we detected a direct correlation between the body weight of the mice and the tumor load measured histologically (**Figure 3F**). Moreover, we found a direct correlation between the tumor load and IGF1R mRNA expression levels of total lung cells (**Figure 3G**).

Altogether, these data indicated that carbohydrate deprivation could be involved in slowing down tumor development leading to less tumor load *in vivo*. As the experimental food also had a different composition regarding fat and protein compared to the high carbohydrate and the breeding diet, effects of fatty acids or other components can´t be excluded. However, consistently to our hypothesis, IGF1R was associated with higher tumor load in the murine model as already seen in the human cohort.

### Carbohydrate Deprivation Induced Tissue-Resident, Central Memory and Effector Memory T Cells and Reduced IL-10 in the Tumor Microenvironment

Carbohydrate deprivation also influences memory T cell homeostasis in tumor-bearing mice. CD8+ memory T cells are heterogeneous in terms of phenotypic molecules and can be classified into central memory T cells (TCM), effector memory T cells (TEM), and tissue-resident memory T cells (TRM). Each subgroup differs in effector functions and tissue-homing capabilities (34). TRM cells are located in tissues and mucosal sites such as the lung and circulate *via* the blood stream. TRM immune cells are important for long-term immunity in their specific tissue. The gating strategy used for this FACS analysis is reported in **Supplementary Figure S4**. The surface molecule CD103 is associated with TRM cells and is essential for their localization close to epithelia (35, 36). TCMs are characterized by CCR7^hi^/CD62L^hi^ expression and stay on location in secondary lymph organs until they get re-activated by antigen presentation (37). Upon antigen encounter, TCMs get activated, and the T cells start rapid proliferation and show differentiation towards effector T cells and migration into other tissues to support immune response. TEMs characterized by CCR7^low^/CD62L^low^ expression are circulating and essential for immune response (38).

**Figure 4:**
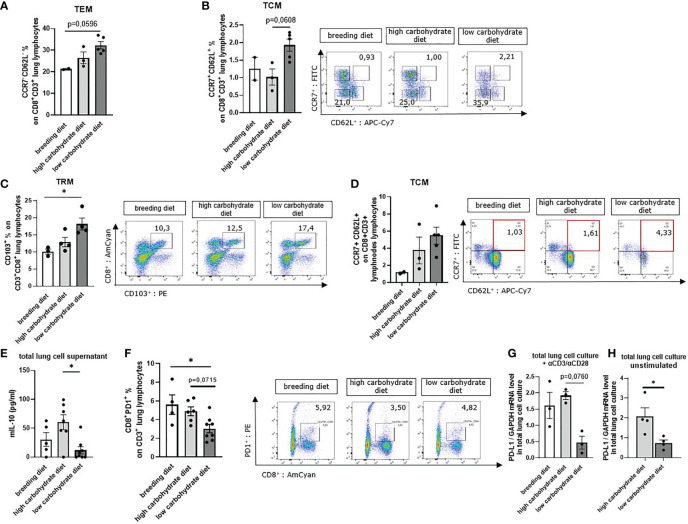
Sugar deprivation induced tissue resident memory T cells (Trm), tissue circulating memory T cells (Tcm) and tissue effector memory T cells (Tem) in mice with LUAD. **(A)** CCR7- CD62L- CD8+ % effector memory T cells (TEM of total lung lymphocytes) gated in total lung cells in mice with LUAD; (n^breeding diet^=2, n^high carbohydrate diet^=3, n^low carbohydrate diet^=5), p (breeding diet vs. low carbohydrate diet) = 0,0596 using Kruskal-Wallis test. **(B)** CCR7+ CD62L+ CD8+ % central memory T cells (TCM) of total lung lymphocytes; (n^breeding diet^=2, n^high carbohydrate diet^=3, n^low carbohydrate diet^=5), p (breeding diet vs. low carbohydrate diet) =0,0608. **(C)** CD103+ CD8+ % tissue-resident memory T cells (TRM) of total lung lymphocytes; (n^breeding diet^=3, n^high carbohydrate diet^=4, n^low carbohydrate diet^=5), p (breeding diet vs. low carbohydrate diet)=0,0255. **(D)** CCR7+ CD62L+ CD8+ % central memory T cells (TCM) of total mesenteric lymph node lymphocytes; (n^breeding diet^=2, n^high carbohydrate diet^=3, n^low carbohydrate diet^=5). **(E)** Murine IL-10 concentration in total lung cell culture supernatant after 5 days cell culture (unstimulated) and analyzed by ELISA; (n^breeding diet^=5, n^high carbohydrate diet^=7, n^low carbohydrate diet^=8), p (high carbohydrate vs. low carbohydrate diet)=0,0340. **(F)** CD3+ CD8+ PD-1+ % T-cells of total lung lymphocytes are shown with representative pseudo color dot plots; (n^breeding diet^=4, n^high carbohydrate diet^=6, n^low carbohydrate diet^=8) p (breeding diet vs. low carbohydrate diet)=0,0268. **(G)** qPCR analysis of PD-L1 mRNA expression level in aCD3/28 stimulated total lung cell culture; (n^breeding diet^=3, n^high carbohydrate diet^ =3, n^low carbohydrate diet^=3), p (high carbohydrate diet vs. low carbohydrate diet)=0,0760. **(H)** qPCR analysis of PD-L1 mRNA expression level in unstimulated total lung cell culture; (n^high carbohydrate diet^ =4, n^low carbohydrate diet^=4), p (high carbohydrate diet vs. low carbohydrate diet) = 0,0286. Kruskal-Wallis test was used for figure **(A–C, E–G)** Mann-Whitney Test was used for **(H)** Data are shown as mean values ± s.e.m.; *P,0.05; of at least three independent experiments.

TRMs can directly control infections but also modulate immune responses to tumors. In addition, secretion of specific cytokines such as IFN-γ can activate dendritic cells and natural killer cells, directly affecting anti-tumoral immunity (39). To understand the effect of a low carbohydrate diet on these T cell subpopulations, we next stained total lung cells with antibodies against CD3, CD8, CD103, CCR7, and CD62L for flow cytometry analyzes. Here we noticed an increase of CD8+CD103+ T cells (TRM) as well as CCR7+CD62L+ (TCM) and CCR7-CD62L- (TEM) cell populations of the CD3+ CD8+ parent population in those mice with low carbohydrate containing food compared to both other groups (**Figures 4A–C**).

To demonstrate an effect on TCMs in lymph nodes, we prepared mesenteric lymph node cells and stained dissociated cells with antibodies against CD3, CD8, CCR7 and CD62L. Here we found increased TCM populations in mice fed with low carbohydrate diet (**Figure 4D**). We next measured IL-10 cytokine concentration in total lung cell supernatant, as IL-10 is a protumoral factor produced by T cells which supports Treg. We found decreased IL-10 concentrations in the cell cultures with glucose starvation compared to those containing glucose in the supernatant (**Figure 4E**).

The programmed cell death protein 1 (PD-1) is one of the most important inhibitory receptors leading to T cell exhaustion after engagement with programmed cell death ligand 1 (PD-L1). T cells expressing high levels of PD-1 on their cell surface mostly lose the ability for effective anti-tumoral immune response. In experimental models, blocking PD-1 receptor partially reversed T cell exhaustion, resulting in enhanced T cell function (40). Therefore, we investigated whether carbohydrate restrictions in the diet influences PD-1 expression on tumor infiltrating CD8+ T cells. In flow cytometric analysis of PD-1 expression on lung CD8+ T cells, we found less PD-1 surface expression in the group with carbohydrate deprivation compared to those mice fed with carbohydrate containing food (**Figure 4F**). We next wanted to investigate the impact of carbohydrate restriction on PD-L1. Thus, we cultured total lung cells for 5 days with RPMI 0 mg/dl glucose and RPMI 200 mg/dl glucose (**Table S5**). Additionally, cells were either stimulated with αCD3/αCD28 antibodies or left untreated. After 5 days, cells were collected, and mRNA expression levels of PD-L1 were analyzed by qPCR. Here, we detected decreased PD-L1 mRNA expression levels in mice with the low carbohydrate diet compared to the carbohydrate containing diet (**Figures 4G, H**).

In conclusion, low carbohydrate diet induces TCM, TEM and TRM T cell populations in mice with experimental LUAD. Moreover, reduced IL-10 levels were detected. These results point out that mice fed with low carbohydrate diet have a better T cell response than mice fed with glucose containing diet. Additionally, we detected decreased PD-1 expression on CD8+ T cell surface in mice fed under carbohydrate restriction, as well as reduced PD-L1 mRNA expression level in total lung cell culture under glucose starvation conditions.

### Carbohydrate Deprivation in the Diet Increased Tumor Infiltrating CD8+ T and Natural Killer Cells and Leads to Increased Anti-Tumoral Cytotoxicity

A major role of CD4+ T cells is providing help for anti-tumor cytotoxic T cells through direct and indirect mechanisms. CD4+ T cells that are activated, secrete interleukin (IL)-2, which directly activates CD8+ CTLs by driving their effector function, differentiation, and proliferation (41). In addition, this CD4+ T cell subset also produces cytotoxic cytokines such as IFN-γ and TNF and therefore directly targets tumor cells (41–43). Natural killer (NK) cells are another important player of anti-tumor immune response and can directly kill tumor cells and influence anti-tumor behavior of other immune cells (44).

**Figure 5:**
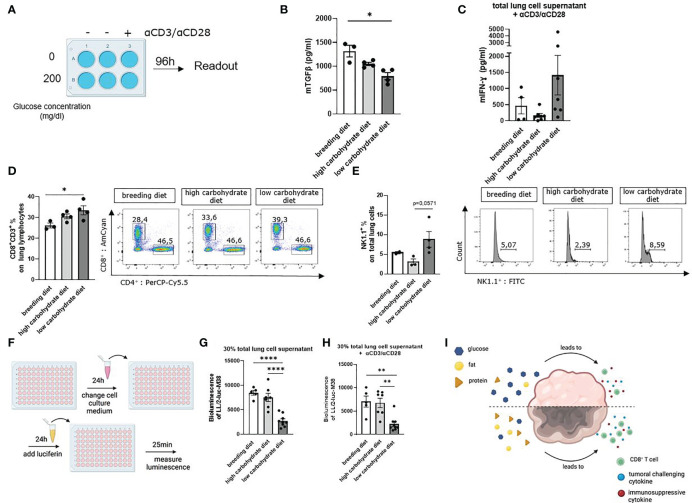
Carbohydrate deprivation in the diet increased tumor infiltrating CD8+ T and natural killer cells and leads to increased anti-tumoral cytotoxicity. **(A)** Schematic illustration of the experimental design. Isolated murine total lung cells were cultured for 96h with 0mg/dl glucose or 200mg/dl glucose, depending on the mice´s diet. Cells from mice fed with carbohydrate containing diet were cultured with 200 mg/dl glucose in the cell culture medium. Cells from mice fed with the low carbohydrate diet were cultured with 0 mg/dl glucose in the cell culture medium. Additionally, cells were either left unstimulated or treated with αCD3/αCD28. **(B)** TGFβ concentration in unstimulated total lung cell culture supernatant, cultured as described in figure A; (n^breeding diet^=3, n^high carbohydrate diet^=4, n^low carbohydrate diet^=4), p (breeding diet vs. low carbohydrate diet) =0,0210. **(C)** Murine IFN-γ concentration in total lung cell culture supernatant, cultured as described in figure A with αCD3/αCD28 treatment. **(D)** CD3+ CD8+ T cell population of total lung cells analyzed by flow cytometry; (n^breeding diet^=3, n^high carbohydrate diet^=4, n^low carbohydrate diet^=4), p (breeding diet vs. low carbohydrate diet) =0,0447. **(E)** NK1.1+ (%) of total lung cells in 24 h cell culture after stimulation with αCD3/αCD28; (n^breeding diet^=2, n^high carbohydrate diet^=3, n^low carbohydrate diet^=4), p (high carbohydrate vs. low carbohydrate diet) =0,0571. **(F)** Schematic illustration of bioluminescent cytotoxicity assay. 7 x 10^3^ LL/2-luc-M38 LUAD cells were cultured in 100 µl for 24 h in a 96 well plate. Supernatant was than discarded and cells were cultured with 30% supernatant of the 5 days total lung cell culture and 70% DMEM medium without additions. After another 24h of culturing cells, supernatant was discarded, and Luciferin was added and incubated for 25 minutes. Bioluminescence was measured with Centro XS3 LB 960. **(G)** Bioluminescence assay with LL/2-luc-M38 LUAD tumor cells, culturing cells with untreated cell culture supernatant of total lung cell culture for 24h; (n^breeding diet^=5, n^high carbohydrate diet^=7, n^low carbohydrate diet^=8), p (breeding diet vs. low carbohydrate diet)=<0,0001, p (high carbohydrate vs. low carbohydrate diet)=<0,0001. **(H)** Bioluminescence assay with LL/2-luc-M38 LUAD tumor cells, culturing cells with αCD3/αCD28 stimulated cell culture supernatant of total lung cell culture for 24 h; (n^breeding diet^=5, n^high carbohydrate diet^=7, n^low carbohydrate diet^=8), p (A vs. C) (breeding diet vs. low carbohydrate diet)=<0,0020, p (high carbohydrate vs. low carbohydrate diet)=<0,0030. **(I)** Schematic representation showing the effect of different nutrition on the TME. One-way ANOVA test was used for figure **(G, H)** Kruskal-Wallis test was used for figure **(B–D)** Mann-Whitney test was used for figure **(E)** Data are shown as mean values ± s.e.m.; *P,0.05; **P,0.01, ****P,0.0001 of at least three independent experiments.

To better understand the effect of glucose on cytokine production, we first analyzed total lung cells in cell culture supernatant *via* ELISA. Therefore, total lung cells were cultured for five days with RPMI 0 mg/dl glucose or RPMI 200 mg/dl glucose, depending on the diet of the mice. Total lung cells from mice fed with the low carbohydrates diet were cultured with RPMI 0 mg/dl glucose. Total lung cells of mice with the high carbohydrate diet and breeding diet were cultured with RPMI 200 mg/dl glucose (**Table S5**). Cells were either stimulated with αCD3/αCD28 antibodies or left untreated (**Figure 5A**). By ELISA measurement of the supernatant from the unstimulated cell cultures, we detected decreased TGF-beta1 concentration upon cultivation without glucose compared to those cultured with 200 mg/dl glucose (**Figure 5B**).

Furthermore, IFN-γ was measured by ELISA in the cell culture supernatants. Here we demonstrated that cells cultured with additional αCD3/αCD28 stimulation and in absence of glucose showed increased IFN-γ concentration in the supernatant compared to the culture containing additional glucose (**Figure 5C**). To understand how carbohydrate deprivation impacts tumor infiltrating CD8+ T cells, we analyzed total CD8+ T cells of the lung by flow cytometry. Here we found an increased infiltrating CD8+ T cell population of mice fed with a low carbohydrate diet (**Figure 5D**). A 24h cell culture of total lung cells, cultured as described above and with additional stimulation with αCD3/αCD28, showed an increase of NK1.1+ cell population in the cell culture without glucose (**Figure 5E**). To simulate a functional tumor microenvironment and investigate the functionality of the lung cell culture supernatant, we performed a bioluminescence cytotoxic assay. Therefore, we challenged LUAD LL/2-luc-M38 cells with the supernatant of total lung cell cultures. As a reporter of surviving LL/2-luc-M38 cells the luciferin mediated bioluminescence reaction was applied (**Figure 5F**). Due to its conversion by luciferase expressed in living LL/2-luc-M38 cells, a highly induced luminescence signal represents higher numbers of living cells. Here we detected decreased luminescence of LL/2-luc-M38 cell culture incubated with unstimulated total lung cell culture supernatant originating from mice fed with the low carbohydrate diet (**Figure 5G**). Similar results were demonstrated after incubation of LL/2-luc-M38 cells with supernatant from the cell culture treated with αCD3/αCD28 antibodies (**Figure 5H**), indicating decreased living LL/2-luc-M38 lung tumor cell numbers in the low carbohydrate condition. These results indicate that carbohydrate deprivation leads to a more induced anti-tumoral immune response in terms of induced cytokine production and induced T cell response (**Figure 5I**).

## Discussion

Therapeutic dietary interventions targeting glucose metabolism are promising to directly inhibit tumor cell development (45). Cycles of fasting and fasting mimicking diets have recently been demonstrated to improve current immunotherapies and to increase resistance to chemotherapy of normal cells but not cancer cells, resulting in prevention of side effects of these aggressive treatments (46, 47). Oncogenic signaling pathways, such as maintenance of DNA replication and several non-mitochondrial pathways, mainly depend on rapid energy availability. Therefore, increased glucose uptake and glycolysis allow tumor cells to maintain large pools of glycolytic intermediates. For that reason, rapid tumor cell progression stays in context with elevated glucose levels in the tumor microenvironment (48, 49). Based on these findings, we analyzed in the present study the functional role of glucose in patients with LUAD and an experimental mouse model of LUAD.

The function of IGF1R and its downstream signaling in tumor cells is still incompletely understood. However, IGFs play an important role in tissue growth (50), glucose homeostasis (33), tumor cell survival and the formation of metastasis. Especially the upregulation of SLUG leads to an activation of other effector proteins, e.g. E-cadherin, Occludin and Vimentin that are essential for cell polarity and adhesion. Furthermore, they influence cytoskeletal reorganization, which is responsible for the formation of metastasis (51, 52).

In the present study, we determined the effects of carbohydrates in the diet, glucose in the TME and the effects of IGF1R on tumor cell growth and progression. To better understand the effects of western pattern diet on LUAD, we analyzed a cohort of patients with histologically confirmed LUAD and specified the findings with *in vitro* human cell culture experiments. Finally, we used a murine model of disease to further explore LUAD progression and T cell immunity *in vivo*.

To analyze our human data, we used the BMI as indicator for body fatness (53) and a retrospective indicator for food intake. We found a direct correlation between the BMI as well as the tumor diameter and IGF1R mRNA expression level. Moreover, a correlation between tumor progression markers (mTOR) and the BMI was observed. In the tumoral and peritumoral region, we found elevated IGF1R mRNA expression levels compared to the control area. Thus, we hypothesize that IGF1R is metabolically and nutrition dependently regulated in the tumoral and peritumoral region of the lung.

In KMplot analysis of blood glucose levels and IGF1R mRNA expression we confirmed a trend for a better 3-year survival of patients with low blood glucose level and low IGF1R mRNA expression. These results were confirmed using Cancer Genome Atlas Program (TCGA) and KMplot data analysis of more than 2000 patients. Here, reduced mRNA expression levels of IGF1R were associated with a significantly better OS in lung cancer patients compared to patients with high IGF1R mRNA expression levels. Therefore, our findings suggest that tumor growth and disease severity are influenced by IGF1R signaling. By analyzing IGF1R expression depending on the tumor grading, we found elevated mRNA levels in patients with G2 grading. We thus concluded that tumor cells, especially in the G2 stage, are sensitized for IGF1, IGF2 and Insulin stimulation by increased glucose in the TME, as these growth hormones can activate IGF1R (54).

Tumor cells show a large variety of immune evasion mechanisms, such as PD-L1 expression and the secretion of effector proteins. Moreover, the tumor is also able to shape the tumor microenvironment by secreting different metabolites and therefore inhibiting antitumoral response. Thus, we hypothesize that increased lactate concentration in cell culture supernatant indicates an upregulation of aerobic glycolytic activity (Warburg effect) due to high glucose in the TME. It is known that high lactate concentrations also contribute to acidification (pH <6.9) of the TME (55), thereby leading to an immune suppressive environment by supporting tumor infiltrating lactate-avid Treg cells (56, 57). In this work, we measured increased lactate concentrations in A549 tumor cell culture supernatant after culturing with increasing glucose concentrations, suggesting that the reduction of glucose in the TME would be beneficial for anti-tumoral immune cells.

Furthermore, we found slightly increased EGFR and drastically increased IGF1R expression on tumor cell surface after elevating the glucose concentration in the cell culture medium. This indicates a strong dependency of IGF1R expression on the glucose concentration in the TME. Previous studies demonstrated an IGF1R/EGFR crosstalk. EGFR tyrosine kinase inhibitors (EGFR TKIs) such as Geftinib, Erlotinib and Afatinib are widely used in treatment of advanced NSCLC (58–60). Thus, we hypothesize that EGFR antibody treatments’ efficacy is reduced by elevated glucose concentrations in the TME through induced IGF1R expression. Treating A549 LUAD cells with IGF1, prevents tumor cell death, induces SLUG expression and stimulates cell migration (**Supplementary Figures S1F–H**). Interestingly, effects of IGF1 are more pronounced, when culturing tumor cells with higher extracellular glucose levels. These results suggest that tumor cells are sensitized to be stimulated with IGFs in higher extracellular glucose in terms of increased IGF1R density.

These results on the effect of glucose and IGF1 confirm the findings and hypothesis of our human study. Our *in vitro* experiments specify the connection between IGF1R expression, tumor progression and the extracellular glucose level. It also demonstrates the potential of IGF1 to support the formation of metastasis. Previous studies showed affected IGF levels, tumor growth and immune response by nutrition intake and dietary modification, such as fasting-mimicking diets (49). Therefore, we assume that dietary modification towards less carbohydrate intake is worth to be considered for patients with LUAD. Directly targeting glucose metabolism by decreasing the extracellular glucose concentration in the TME could induce the anti-tumoral immune response. It might repress the activity of lactate avid Treg cells and even support immune checkpoint therapy, such as anti-PD-1/anti-PD-L1 treatment in LUAD. These results imply the need to consider anti-IGF1R treatment to support current anti-tumoral therapy. However, whether carbohydrate restriction as a diet is beneficial in supporting immunotherapy and anti-tumor response remains unknown and needs to be addressed by controlled clinical trials.

To investigate the effect of carbohydrate restriction on LUAD under *in vivo* conditions, we took advantage of a murine model of LUAD. Interestingly, we found a direct correlation between the body weight of the mice and the tumor load, as well as a trend of elevated IGF1R mRNA expression levels correlating with increased tumor load. Moreover, we found a reduction of total tumor load in mice fed with a low carbohydrate diet. These mice also showed better OS and enhanced anti-tumoral immunity, as shown by increased IFN-γ and reduced IL-10 production. These findings in our murine model confirmed the results of our human study and our cell culture experiments. They point out, that the IGF1R signaling axis influences tumor development in a progressive manner.

Secreted IFN-γ has various synergistic effects on the immune system. It increases and prolongs the expression of antigen presentation *via* induction of MHC class I molecules. Furthermore, it enhances NK cells, CTLs activity and induces regulatory T cell fragility (61, 62). However, IFN-γ induces PD-L1 expression in tumor cells, which is one of the most important tumor immune evasion mechanisms in NSCLC. By the interaction of PD-L1 and PD-1 on the T cell surface, T cell cytokine production is inhibited and anti-tumoral immune response is exhausted (63–65). In this context, we found repressed PD-1 expression on CD8+ T cell surface and decreased PD-L1 mRNA expression levels in total lung cell culture of mice fed with low carbohydrate diet.

In further analyses, we measured the immune-suppressive cytokine TGFβ in the cell culture supernatant. Here we found decreased TGFβ concentration in the unstimulated low carbohydrate diet group. In summary, the cytokine analysis of total lung cell culture suggested increased anti-tumoral T cell response under carbohydrate deprivation.

Most importantly, in a cytotoxicity bioluminescence assay performed with the cell culture supernatant, we demonstrated decreased tumor cell numbers in the low carbohydrate group. These data indicate a tumor challenging TME because of increased anti-tumoral immunity due to cytokine production by immune cells and targeting tumor cell metabolism by glucose starvation. This was demonstrated in cell cultures under glucose starvation in an unstimulated condition and upon αCD3/αCD28 stimulation.

Despite these new findings, limitations and improvements of used methods have to be discussed. In our murine model of LUAD, mice fed with low carbohydrate diet showed initial rapid weight loss. On day 4, this group of mice gained weight up to their initial weight. Interestingly, these mice appeared more vital during the whole time, indicating less disease severity, which is consistent with the tumor load detection. As glucose restricted diet challenged the mice, we used infrared light (IR) to create an alternative energetic support. Therefore, all mice were kept under IR, which induced the environmental temperature. Assuming that IR is able to reach the mouse body tissues, it will induce preferentially tumor cell death, as cancer tissues are more thermosensitive than normal tissues due to their acidic interstitial environment, reduced heat dissipation capacity and increased metabolic stress (66). This will result in an induced anti-tumor microenvironment. However, all groups of mice were exposed to IR light in the same manner for short, defined intervals, excluding the possibility that IR light is responsible for the observed effects of low versus high carbohydrate diets.

In our model of disease, we changed the food two days before tumor initiation. Therefore, it is not possible to determine the therapeutical value of low carbohydrate diet in mice with established tumors. However, the findings in our model unequivocally demonstrate that decreasing glucose concentration in the diet immediately before tumor injection reduces the development of LUAD in mice in a prophylactic setting.

As the low carbohydrate diet contains higher protein and higher fat concentration compared to both other diets, we cannot conclude that low carbohydrate concentration in the diet is the only factor challenging tumor development. In addition, increased protein and fat levels in the low carbohydrate diet might affect the tumor development. Both protein and fat, might also challenge tumor growth and support immune response. Moreover, the low carbohydrate diet we used is hypocaloric, containing 1.394 kcal/kg. The control diet contains 3.506 kcal/kg and the breeding diet 3.466 kcal/kg. During the experiment we monitored the food intake of all groups and found an increased uptake in the low carbohydrate diet group, which compensated for the reduced energy in the food and therefore in average, all mice had similar energy intake.

In conclusion, we demonstrated that IGF1R mRNA expression level directly correlates with the BMI and tumor diameter of patients with LUAD. IGF1R as well as increased blood glucose levels were associated with worse OS. Reducing the extracellular glucose concentration led to less tumor cell proliferation and less growth hormone receptor expression, measured on protein level by flow cytometry. IGF1 treatment in a LUAD cell line caused increased glucose consumption and higher GLUT4 expression. In the murine model of LUAD, we demonstrated decreased total tumor load, increased OS and an increased anti-tumoral T cell response by carbohydrate deprivation. Moreover, we noticed a correlation between the tumor load and the weight as well as IGF1R mRNA expression. Previous studies already highlighted the potential of dietary modification as additional setting to chemotherapy. Fasting or fasting mimicking diets (FMDs) can prevent DNA damage in healthy cells caused by chemotherapy and maintain relapse-free survival (46). Other studies dealt with increased metabolic vulnerability in cisplatin resistant NSCLC cells (47). Regarding these findings, our study offers the potential of carbohydrate restricted diet as additional setting to chemotherapy, and cisplatin resistant LUAD cell treatment.

In summary, our study enlightens the potential of IGF1R and the downstream signaling as a target for anti-tumoral therapy. Moreover, we demonstrated that glucose metabolism affects growth of lung tumor cells and regulates the lung immune microenvironment by controlling effector memory and cytotoxic T cell responses. The translational relevance of our findings is underlined by studies in LUAD patients showing reduced survival rates after surgery in the presence of high glucose levels. As glucose levels in LUAD patients were negatively correlated to postoperative survival rates, glucose-restricted diet emerges as potentially attractive therapeutic concept for patients with lung adenocarcinoma.

## Author’s Note

The present work was performed in fulfilment of the requirements for obtaining the degree “Dr. med.” for Alexander Gähler.

## Data Availability Statement

The datasets generated for this study can be accessed upon request to the corresponding author.

## Ethics Statement

The studies involving human participants were reviewed and approved by Ethics review board of the University of Erlangen-Nürnberg (Re-No: 56_12B). The patients/participants provided their written informed consent to participate in this study. The animal study was reviewed and approved by Regierung Unterfranken (Az 55.2-2532-2-1286-20).

## Author Contributions

AG performed all the murine and cell culture experiments. He analyzed all the data and wrote the first draft of the manuscript. DT and HS are the thoracic surgeons who recruited the NSCLC patients and performed the lung tumor resection surgery. MC helped with the murine model of lung cancer. PT and KH helped with the human RNA isolation, FACS analysis, cell culture, edited the manuscript and contributed to revision. MR measured the glucose and lactate levels in the supernatant of different samples. CG and RR coordinated the histology and performed pathologic analysis presented in this manuscript. A-KB and AL helped with the murine experiments, analysis and interpretation of the data. SK helped with data selection and analysis and contributed to the writing of the manuscript. MN provided help with editing the manuscript. HS and AH coordinated the study cohort on NSCLC patients with SF. SF supervised the entire work, contributed to analysis and interpretation of the data and contributed to the writing of the manuscript. All authors contributed to the article and approved the submitted version.

## Funding

This work was supported by the Deutsche Forschungsgemeinschaft (DFG or German Research Foundation) FI 817- 5/1-3, the CRC1181 or SFB1181 – 261193037 Project B08N in Erlangen, a Wilhelm Sander-Stiftung grant (Project number 2020.016.1.) and by the Department of Molecular Pneumology at the FAU Uniklinikum Erlangen.

## Conflict of Interest

The authors declare that the research was conducted in the absence of any commercial or financial relationships that could be construed as a potential conflict of interest.

## Publisher’s Note

All claims expressed in this article are solely those of the authors and do not necessarily represent those of their affiliated organizations, or those of the publisher, the editors and the reviewers. Any product that may be evaluated in this article, or claim that may be made by its manufacturer, is not guaranteed or endorsed by the publisher.
