# Supplementary material for: Glucose-Restricted Diet Regulates the Tumor Immune Microenvironment and Prevents Tumor Growth in Lung Adenocarcinoma
Source: Front Oncol. 2022 Apr 29;12:873293. doi: 10.3389/fonc.2022.873293 (PMC9102798; doi:10.3389/fonc.2022.873293)
Supplement: Supplementary file 1 [file DataSheet_1.pdf]

# *Supplementary Material*

# Glucose-restricted diet regulates the tumor immune microenvironment and prevents tumor growth in lung adenocarcinoma

**Alexander Gähler[1,**], Denis I. Trufa[2], Mircea T. Chiriac[3], Patrick Tausche[1,┼], Katja Hohenberger[1,┼], Ann-Kathrin Brunst[1], Manfred Rauh[4], Carol I. Geppert [5], Ralf J. Rieker[5,6], Susanne Krammer[1], Anna Leberle[1], Markus F. Neurath[3], Horia Sirbu[2,7], Arndt Hartmann[5,6], and Susetta Finotto [1,6,*]**

[1] Department of Molecular Pneumology, Friedrich-Alexander-Universität (FAU) Erlangen-Nürnberg, Universitätsklinikum Erlangen, 91052 Erlangen, Germany

[2] Department of Thoracic Surgery, Friedrich-Alexander-Universität (FAU) Erlangen-Nürnberg, Universitätsklinikum Erlangen, 91052 Erlangen, Germany

[3] Department of Medicine 1 - Gastroenterology, Pneumology and Endocrinology, Friedrich-Alexander-Universität (FAU) Erlangen-Nürnberg, Universitätsklinikum Erlangen, 91052 Erlangen, Germany

[4] Department of Paediatrics and Adolescent Medicine, Friedrich-Alexander-Universität (FAU) Erlangen-Nürnberg, Universitätsklinikum Erlangen, 91052 Erlangen, Germany

[5] Institute of Pathology, Friedrich-Alexander-Universität (FAU) Erlangen-Nürnberg, Universitätsklinikum Erlangen, 91052 Erlangen, Germany

[6] Comprehensive Cancer Center Erlangen-EMN (CCC ER-EMN), Erlangen, Germany

**The present work was performed in fulfilment of the requirements for obtaining the degree "Dr. med." for Alexander Gähler

┼Contributed equally to this work

*** Correspondence:**
Prof. Susetta Finotto, PhD

Department of Molecular Pneumology

Friedrich-Alexander-Universität Erlangen-Nürnberg

Hartmannstraße 14

91052 Erlangen, Germany

## 1. Supplementary Tables

**Supplementary Table S1: Clinical data of the cohort of patients with NSCLC analyzed in this study**

| Patient Code | Histological classification | Grading | Age | Gender | Tumor Diameter (cm) | pT | pN | M | TNM-Stadium | BMI = Weight/ Hight² | IGF1R Expression TU | glucose (mg7dl) |
|---|---|---|---|---|---|---|---|---|---|---|---|---|
| 3-MP | ADC | G3 | 79 | Male | 5 | 2b | 0 | 0 | IIA | 27,44 | - | - |
| 9-MP | ADC | G2 | 84 | Female | 2,7 | 1c | 2 | 0 | IIIA | 30,04 | - | 83 |
| 10-MP | ADC | G2 | 72 | Male | 1,4 | 1b | 0 | 0 | IA2 | 22,20 | - | - |
| 15-MP | ADC | G3 | 63 | Male | 2,5 | 1c | 0 | 0 | IA3 | 31,38 | - | 125 |
| 16-MP | ADC | G3 | 70 | Female | 1,8 and 2,8 | 3 | 0 | 0 | IIB | 27,68 | - | 107 |
| 17-MP | ADC | G2 | 74 | Male | 2,6 | 1c | 0 | 0 | IA3 | 30,42 | - | 110 |
| 19-MP | ADC | - | 55 | Female | 7 | 3 | 0 | 0 | IIB | 23,67 | - | |
| 20-MP | ADC | G3 | 60 | Male | 2,7 | 1c | 0 | 0 | IA3 | 21,04 | - | 110 |
| 22-MP | ADC | G3 | 68 | Male | 7 | 3 | 1 | 0 | IIIA | 27,32 | - | 117 |
| 23-MP | ADC | G2 | 73 | Male | 4,5 | 2b | 0 | 0 | IIA | 23,55 | - | 101 |
| 26-MP | ADC | G3 | 52 | Female | 1,3 | 1b | 0 | 1b | IVA | 28,52 | - | 104 |
| 27-MP | ADC | G3 | 70 | Female | 1,4 | 1b | 0 | 0 | IA2 | 26,40 | - | 93 |
| 28-MP | ADC | G3 | 76 | Male | 1,2 | 1b | 0 | 0 | IA2 | 28,40 | - | 100 |
| 32-MP | ADC | G3 | 60 | Female | 4,4 | 2b | 2 | 0 | IIIA | 26,44 | - | |
| 34-MP | ADC | G2 | 51 | Female | 1,8 | 1b | 0 | 0 | IA2 | 20,20 | - | 84 |
| 35-MP | ADC | G3 | 72 | Female | 3 | 1c | 0 | 0 | IA3 | 35,56 | - | 102 |
| 39-MP | ADC | G3 | 65 | Male | 6 | 3 | 0 | 0 | IIB | 33,66 | - | 134 |
| 40-MP | ADC | G3 | 82 | Male | 1,8 | 1b | 1 | 0 | IIB | 17,65 | - | 86 |
| 43-MP | ADC | G3 | 72 | Female | 4 | 2a | 2 | 0 | IIIA | 30,85 | - | 96 |
| 44-MP | ADC | G1 | 53 | Male | 1,5 | 1b | 0 | 0 | IA2 | 34,32 | - | 108 |
| 45-MP | ADC | G1 | 78 | Male | 2,3 | 1c | 0 | 0 | IA3 | 35,43 | - | 94 |
| 51-MP | ADC | G3 | 61 | Male | 2,3 and 2,5 | 3 | 2 | 0 | IVA | 24,07 | - | 96 |
| 53-MP | ADC | G2 | 62 | Male | 1,4 | 1b | 0 | 0 | IA2 | 30,98 | - | 101 |
| 55-MP | ADC | G3 | 64 | Female | 1,8 | 1b | 2 | 0 | IIIA | 29,30 | - | 100 |
| 56-MP | ADC | G2 | 68 | Female | 4 | 2a | 0 | 0 | IB | 40,40 | - | 92 |
| 57-MP | ADC | G2 | 36 | Female | 3,8 | 2a | 0 | 0 | IB | 49,25 | - | 112 |
| 59-MP | ADC | G2 | 71 | Male | 0,9 and 2,1 | 4 | 0 | 0 | IIIA | 21,47 | - | 100 |
| 62-MP | ADC | G2 | 80 | Female | 3,5 | 2a | 0 | 0 | IB | 45,91 | - | 161 |

| | | | | | | | | | | | | |
|---|---|---|---|---|---|---|---|---|---|---|---|---|
| 64-MP | ADC | G2 | 55 | Male | 3,5 | 2a | 1 | 1a | IVA | 24,93 | - | 89 |
| 68-MP | ADC | G3 | 42 | Male | 8,5 | 4 | 0 | 0 | IIIA | 26,47 | 4,026 | 94 |
| 69-MP | ADC | G2 | 76 | Female | 3,1 | 2a | 0 | 0 | IB | 25,24 | - | 81 |
| 70-MP | ADC | G2 | 70 | Female | 2,7 | 1c | 0 | 0 | IA3 | 22,10 | - | - |
| 71-MP | ADC | G3 | 67 | Male | 3,7 | 2a | 0 | 0 | IB | 30,42 | - | 109 |
| 72-MP | ADC | - | 53 | Male | 3,5 | | | | IIIC | 24,98 | - | 104 |
| 73-MP | ADC | G2 | 67 | Female | 4,8 | 2b | 0 | 0 | IIA | 27,36 | - | 93 |
| 74-MP | ADC | G2 | 58 | Female | 3,2 | 2a | 0 | 0 | IB | 30,73 | - | - |
| 77-MP | ADC | G2 | 64 | Female | 0,9 | 1a | 0 | 0 | IA1 | 24,09 | - | 259 |
| 78-MP | ADC | G3 | 80 | Female | 2,1 | 1c | 2 | 0 | IIIA | 26,29 | - | 102 |
| 80-MP | ADC | G3 | 62 | Male | 5,4 | 3 | 0 | 0 | IIB | 34,87 | - | 106 |
| 81-MP | ADC | G3 | 61 | Male | 1,6 | 1b | 0 | 0 | IA2 | 26,09 | - | 98 |
| 83-MP | ADC | G3 | 60 | Female | 5,5 | 3 | 0 | 1a | IVA | 23,42 | - | 98 |
| 86-MP | ADC | G2 | 54 | Male | 1,8 | 1b | 1 | 1b | IVA | 17,76 | - | 65 |
| 88-MP | ADC | G2 | 73 | Male | 2,1 | 1c | 0 | 0 | IA3 | 25,61 | - | 102 |
| 89-MP | ADC | - | 61 | Female | 3,7 | 2a | 2 | 0 | IIIA | 24,88 | - | 94 |
| 91-MP | ADC | G2 | 67 | Male | 3,6 | 2a | 2 | 0 | IIIA | 24,06 | 0,44 | 101 |
| 93-MP | ADC | G3 | 66 | Male | 2,8 | 2a | 1 | 0 | IIB | 29,07 | - | 104 |
| 94-MP | ADC | G2 | 77 | Female | 2,2 | 1c | 0 | 0 | IA3 | 23,57 | - | 108 |
| 97-MP | ADC | G2 | 73 | Male | 5 | 3 | 0 | 0 | IIB | 29,14 | 1,999 | 127 |
| 98-MP | ADC | G3 | 55 | Female | 2,9 | 1c | 2 | 0 | IIIA | 17,22 | 0,127 | 102 |
| 99-MP | ADC | G3 | 62 | Male | 3,5 and 1,5 | 4 | 0 | 0 | IIIA | 18,93 | 1,034 | - |
| 102-MP | ADC | G2 | 66 | Male | 2,1 | 1c | 1 | 0 | IIB | 20,90 | 0,281 | 101 |
| 103-MP | ADC | G3 | 70 | Female | 3,9 | 2a | 0 | 0 | IB | 24,52 | 0,806 | 108 |
| 105-MP | ADC | G3 | 77 | Male | 1,8 | 1b | 0 | 0 | IA2 | 25,86 | 1,288 | - |
| 106-MP | ADC | G2 | 79 | Male | 1,2 | 1b | 0 | 0 | IA2 | 24,45 | 1,191 | - |
| 107-MP | ADC | G1 | 54 | Female | 1,7 | 1b | 0 | 0 | IA2 | 21,48 | 0,71 | 97 |
| 108-MP | ADC | G2 | 60 | Female | 3,6 | 2a | 0 | 0 | IB | 20,03 | 1,015 | 116 |
| 110-MP | ADC | G3 | 77 | Female | 7,2 | 4 | 2 | 0 | IIIB | 25,64 | 2,371 | - |
| 112-MP | ADC | G2 | 74 | Female | 1,6 | 4 | 0 | 0 | IIIA | 29,36 | 2,577 | 101 |
| 113-MP | ADC | G3 | 68 | Male | 7,5 | 3 | 0 | 0 | IIB | 27,77 | - | 166 |
| 118-MP | ADC | G1 | 67 | Female | 1,5 | 1b | 0 | 0 | IA2 | 33,25 | 1,4 | - |
| 121-MP | ADC | G3 | 59 | Male | 12,5 | 4 | 0 | 0 | IIIA | 25,00 | - | - |
| 122-MP | ADC | G3 | 61 | Female | 2,8 | 1c | 0 | 0 | IA3 | 37,03 | - | 94 |
| 124-MP | ADC | G2 | 71 | Male | 1,2 | 1b | 0 | 0 | - | - | - | - |

Abbreviations: MP= molecular pneumology, ADC=adenocarcinoma; BMI= body mass index

Histopathological grading: G1=well differentiated; G2=moderately differentiated; G3=poorly differentiated

T-primary tumor: 0: No evidence of primary tumor; 1a: Tumor 2 cm or less in greatest dimension; 1b: Tumor more than 2 cm but not more than 3 cm in greatest dimension; 2a: Tumor more than 3 cm but not more than 5 cm in greatest dimension; 2b: Tumor more than 5 cm but not more than 7 cm in greatest dimension; 3: Tumor more than 7 cm
N-regional lymph nodes: 0: No regional lymph node metastasis; 1: Metastasis in ipsilateral peribronchial and/ or ipsilateral hilar lymph nodes and intrapulmonary nodes, including involvement by direct extension; 2: Metastasis in ipsilateral mediastinal and/or subcarinal lymph node(s)
M-distant metastasis: 0: No distant metastasis; 1: Distant metastasis.

**Supplementary Table S2: Summary of relevant clinical data of the cohort of patients with NSCLC analyzed in this study**

| | | |
|---|---|---|
| Number of Patients | 63 | |
| Male | 33 (52,4%) | |
| Female | 30 (47,6%) | |
| Av. Age | 65,98412698 | (σ=9,704) |
| G1 | 3 | |
| G2 | 26 | |
| G3 | 30 | |
| tumor diameter | 3,42 | (σ=2,164) (n=63) |
| Av. BMI | 27,36 | (σ=6,157) (n=62) |
| Av. Glucose | 106,7 | (σ=27,753) (n=51) |

**Supplementary Table S3: Survival data of the cohort of patients with NSCLC analyzed in this study**

| Patient Code | Survival after 3 months (CT) | Survival after 6 months (CT) | Survival after 9 months (CT) | Survival after 12 months (CT) | Survival after 3 years (CT) | Survival after 5 years (CT) |
|---|---|---|---|---|---|---|
| 3-MP | No | | | | | |
| 9-MP | yes | yes | yes | yes | yes | yes |
| 10-MP | yes | yes | yes | yes | yes | |
| 15-MP | yes | | | | | |
| 16-MP | yes | yes | yes | | | |
| 17-MP | yes | yes | yes | yes | yes | yes |
| 19-MP | | | | | | |
| 20-MP | yes | yes | | | | |
| 22-MP | yes | yes | yes | yes | yes | |
| 23-MP | yes | yes | yes | yes | | |
| 26-MP | | | | | | |
| 27-MP | yes | yes | yes | yes | yes | yes |
| 28-MP | | | | | | |
| 32-MP | yes | yes | yes | yes | | |
| 34-MP | yes | yes | yes | yes | yes | yes |
| 35-MP | yes | yes | yes | yes | yes | yes |
| 39-MP | yes | yes | yes | yes | | |
| 40-MP | yes | yes | yes | yes | | |
| 43-MP | | | | | | |
| 44-MP | yes | yes | yes | yes | | |
| 45-MP | | | | | | |
| 51-MP | yes | yes | yes | yes | yes | |
| 53-MP | yes | yes | yes | yes | | |
| 54-MP | yes | yes | | | | |
| 55-MP | yes | yes | yes | yes | | |
| 56-MP | yes | yes | | | | |
| 57-MP | yes | yes | | | | |
| 59-MP | yes | yes | | | | |
| 62-MP | yes | yes | yes | yes | | |
| 64-MP | yes | yes | yes | yes | yes | |
| 68-MP | yes | yes | yes | yes | | |
| 69-MP | yes | yes | yes | | | |
| 70-MP | | | | | | |
| 71-MP | | | | | | |
| 72-MP | yes | yes | yes | yes | | |
| 73-MP | yes | yes | yes | | | |
| 74-MP | yes | yes | yes | yes | | |
| 77-MP | | | | | | |
| 78-MP | yes | yes | yes | yes | | |
| 80-MP | yes | yes | yes | yes | | |
| 81-MP | yes | yes | yes | yes | | |
| 83-MP | | | | | | |
| 86-MP | yes | yes | yes | yes | | |
| 88-MP | | | | | | |
| 89-MP | yes | yes | yes | yes | | |
| 91-MP | | | | | | |
| 93-MP | yes | yes | yes | | | |
| 94-MP | | | | | | |
| 97-MP | yes | yes | yes | yes | | |
| 98-MP | yes | yes | | | | |
| 99-MP | no | | | | | |
| 102-MP | yes | yes | yes | yes | | |
| 103-MP | | | | | | |
| 105-MP | yes | yes | yes | yes | | |
| 106-MP | yes | yes | yes | yes | | |
| 107-MP | yes | yes | yes | yes | | |
| 108-MP | yes | yes | yes | | | |
| 110-MP | yes | yes | yes | | | |
| 112-MP | yes | yes | yes | | | |
| 113-MP | | | | | | |
| 118-MP | yes | | | | | |
| 121-MP | yes | | | | | |
| 122-MP | yes | | | | | |
| 124-MP | | | | | | |

**Supplementary Table S4: List of primers used for Quantitative Real-Time PCR.**

| GENE | PRIMER SEQUENCE |
|---|---|
| hIGF1R | fw: 5´- GGG GCT CCT GTT TCT CTC C -3´<br>rev: 5´- GCC TTG GAG ATG AGC AGG AT-3´ |
| hAKT | fw: 5´- CAC ACC ACC TGA CCA AGA TGA -3´<br>rev: 5´- CCT CCA AGC TAT CGT CCA GC-3´ |
| hSLUG | fw: 5´- GAA CTG GAC ACA CAT ACA GTG ATT-3´<br>rev: 5´- AGT GAT GGG GCT GTA TGC TC-3´ |
| hmTOR | fw: 5 - AGC ATC GGA TGC TTA GGA GTG G – 3´<br>rev: 5´ - CAG CCA GTC ATC TTT GGA GAC C – 3´ |
| *hGAPDH* | fw: 5´- AAA TCA AGT GGG GCG ATG CT – 3´<br>rev: 5´- CAA ATG AGC CCC AGC CTT CT – 3´ |
| *mPD-L1* | fw: 5´- TCA CTT GCT ACG GGC GTT T – 3´<br>rev: 3´- CCC AGT ACA CCA CTA ACG CA – 5´ |
| *mGAPDH* | fw: 5´- AAC TTT GGC ATT GTG GAA GG– 3´<br>rev: 3´- CAC ATT GGG GGT AGG AAC AC– 5´ |
| mIGFR | fw: 5´- CTG CGG CGA TGA AGA GAA GAA AA – 3´<br>rev: 5´- TAC CGG TGC CAC GTT ATG ATG ATT – 3´ |

**Supplementary Table S5: Cell culture media used for human and mouse experiments**

| MEDIUM | INGREDIENTS |
|---|---|
| RPMI 0 mg/dl glucose | 500 ml Gibco™ RPMI 1640 Medium, Thermo Fisher Scientific, no glucose (cat# 11879020); 50 ml heat-inactivated fetal bovine serum (FCS), Sigma-Aldrich (cat# S0615); 2mM L-Glutamine, anprotec (cat# AC-AS-0001); 5 ml Penicillin-Streptomycin (Pen/Strep), anprotec (cat# AC-AB-0024) |
| RPMI 100 mg/dl glucose | For RPMI 100 mg/dl final medium for 0 mg/dl and 200 mg/dl were mixed 1:1 |
| RPMI 200 mg/dl glucose | 500 ml Gibco™ RPMI 1640 Medium, Thermo Fisher Scientific (cat# 21875091); 50 ml heat-inactivated fetal bovine serum (FCS), Sigma-Aldrich (cat# S0615); 2mM L-Glutamine, anprotec (cat# AC-AS-0001); 5 ml Penicillin-Streptomycin (Pen/Strep), anprotec (cat# AC-AB-0024) |
| DMEM medium | 500 ml Gibco™ DMEM, high glucose, Thermo Fisher Scientific (cat# 11965092); 50 ml heat-inactivated fetal bovine serum (FCS), Sigma-Aldrich (cat# S0615); 2mM L-Glutamine, anprotec (cat# AC-AS-0001); 5 ml Penicillin-Streptomycin (Pen/Strep), anprotec (cat# AC-AB-0024) |
| DMEM medium without additions | 500 ml Gibco™ DMEM, high glucose, Thermo Fisher Scientific (cat# 11965092) |

**Supplementary Table S6: List of antibodies used for human flow-cytometry**

| REAGENT or RESOURCE | SOURCE | IDENTIFIER |
|---|---|---|
| Mouse monoclonal anti-human-CD221, BV421 | BD Biosciences | Cat#562593 |
| Mouse monoclonal anti-human-EGFR, AlexaFlour488 | BioLegend | Cat#352907 |
| Annexin V, APC | BD Biosciences | Cat#550474 |
| Propidium Iodide Staining Solution | BD Biosciences | Cat#51-66211E |
| Mouse Anti-Ki-67- FITC | BD Biosciences | Cat#556026 |
| Human BD Fc Block | BD Biosciences | Cat#564220 |

**Supplementary Table S7: List of cell culture antibodies and recombinant proteins**

| Chemicals, peptides, cell culture antibodies and recombinant proteins | | |
|---|---|---|
| Human IGF1 | R&D Systems | Cat#291-G1 |
| Murine aPD1 | InVivoMab | Cat#BE0146 |
| Murine IgG2a | InVivoMab | Cat#BE0089 |
| Murine aCD3 | BD Biosciences | Cat#555329 |
| Murine aCD28 | BD Biosciences | Cat#555725 |
| Murine aCD16/CD32 | BD Biosciences | Cat#553142 |

**Supplementary Table S8: List of antibodies used for murine flow-cytometry**

| REAGENT or RESOURCE | SOURCE | IDENTIFIER |
|---|---|---|
| Monoclonal anti-murin-CD3, FITC | eBioscience | Cat#11-0031-82 |
| Hamster monoclonal anti-murin-CD3, PE-Cy7 | eBioscience | Cat#552774 |
| Rat monoclonal anti-murin-CD4, BB700 | BD Biosciences | Cat#566408 |
| Rat monoclonal anti-murin-CD4, PE-Cy5.5 | BD Biosciences | Cat#550954 |
| Rat monoclonal anti-murin-CD8, BV510 | BD Biosciences | Cat#563068 |
| Monoclonal anti-murin-CD8, PerCP | BioLegend | Cat#100732 |
| Rat monoclonal anti-murin-CD25, BV421 | BioLegend | Cat#102033 |
| Hamster monoclonal anti-murin-PD1, PE | BD Biosciences | Cat#561788 |
| Rat monoclonal anti-murin-CD62L, APC-Cy7 | BD Biosciences | Cat#560514 |
| Rat monoclonal anti-murin-CCR7, AlexaFlour488 | BD Biosciences | Cat#560682 |
| Monoclonal anti-murin-CD103, PE | eBioscience | Cat#12-1031-83 |
| Mouse BD Fc Block | BD Biosciences | Cat# 553142 |

**Supplementary Table S9: Enzyme-linked immunosorbent assay (ELISA)**

| REAGENT or RESOURCE | SOURCE | IDENTIFIER |
|---|---|---|
| ELISA | | |
| Murine IFN-γ | eBioscience | Cat#555138 |
| Murine IL-2 | eBioscience | Cat#555148 |
| Murine IL-10 | BD Bioscience | Cat#555252 |
| Murine TGF-β | R&D system | Cat#DY9679 |

**Supplementary Table S10: Micro nutrition of the mice diet**

| **Diet** | **High carbohydrate diet** | **Low carbohydrate diet** | **Breeding diet** |
|---|---|---|---|
| | | | |
| **Trace elements** | **[mg/kg]** | **[mg/kg]** | **[mg/kg]** |
| Aluminium | 3,71 | 16,03 | 82,46 |
| Chlorine | 3.630,00 | 3.630,00 | 3.369,44 |
| Iron | 178,58 | 179,3 | 189,98 |

| | | | |
|---|---|---|---|
| Flourine | 4,17 | 4,17 | 3,1 |
| Iodine | 0,51 | 0,51 | 1,47 |
| Cobalt | 0,15 | 0,14 | 0,48 |
| Copper | 5,75 | 5,65 | 14,52 |
| Manganese | 100,89 | 101,52 | 72,88 |
| Molybdenum | 0,2 | 0,2 | 1,67 |
| Sulfur | 2.791,54 | 2.712,65 | 918,24 |
| Selenium | 0,33 | 0,31 | 0,26 |
| Zinc | 29,3 | 30,04 | 83,65 |
| | | | |
| **Added vitamins per kg** | | | |
| Biotin | 201 µg | 201 µg | 290 µg |
| Choline chloride | 1.012 mg | 1.012 mg | 717 mg |
| Folic acid | 10 mg | 10 mg | 2 mg |
| Nicotinic acid | 50 mg | 50 mg | 36 mg |
| Pantothenic acid | 50 mg | 50 mg | 21 mg |
| Vitamine A | 15.000 IU | 15.000 IU | 15.000 IU |
| Vitamine B1 | 20 mg | 20 mg | 18 mg |
| Vitamine B2 | 20 mg | 20 mg | 12 mg |
| Vitamine B6 | 15 mg | 15 mg | 9 mg |
| Vitamine B12 | 41 µg | 41 µg | 24 µg |
| Vitamine C | 20 mg | 20 mg | 36 mg |
| Vitamine D3 | 500 IU | 500 IU | 600 IU |
| Vitamine E | 180 mg | 192 mg | 80 mg |
| Vitamine K3 | 10 mg | 10 mg | 3 mg |
| | | | |
| **Fatty acids** | **[mg/kg]** | **[mg/kg]** | **[mg/kg]** |
| Arachidic acid C-20:0 | 50 | 70 | 340 |
| Eicosanoic acid C-20:1 | 150 | 210 | 425 |
| α-Linolenic acid C-18:3 | 150 | 210 | 4.491 |
| Linolenic acid C-18:2 | 28.500 | 39.900 | 32.681 |
| Palmitic acid C-16:0 | 2.500 | 3.500 | 8.010 |

| | | | |
|---|---|---|---|
| Stearic acid C-18:0 | 1.350 | 1.890 | 2.399 |
| Oleic acid C-18:1 | 13.500 | 18.900 | 13.795 |
| | | | |
| **Amino acids** | **[mg/kg]** | **[mg/kg]** | **[mg/kg]** |
| Alanine | 2.528 | 2.316 | 10.395 |
| Arginine | 9.829 | 9.700 | 15.977 |
| Aspartic acid | 3.583 | 3.404 | 23.673 |
| Cystine | 3.196 | 3.140 | 3.285 |
| Glutaminc acid | 23.675 | 23.172 | 46.578 |
| Glycine | 3.136 | 3.030 | 10.055 |
| Histidine | 5.276 | 5.200 | 5.800 |
| Isoleucine | 7.223 | 7.120 | 10.406 |
| Leucine | 14.763 | 14.440 | 17.772 |
| Lysine | 17.401 | 17.322 | 12.372 |
| Methionine | 7.223 | 7.170 | 3.530 |
| Phenylalanine | 7.172 | 7.040 | 11.293 |
| Proline | 12.763 | 12.516 | 14.511 |
| Serine | 5.268 | 5.130 | 12.016 |
| Threonine | 7.154 | 7.054 | 8.758 |
| Tryptophan | 1.977 | 1.960 | 3.192 |
| Tyrosine | 9.285 | 9.170 | 7.953 |
| Valine | 3.296 | 3.170 | 11.405 |

## 2. Supplementary Figures

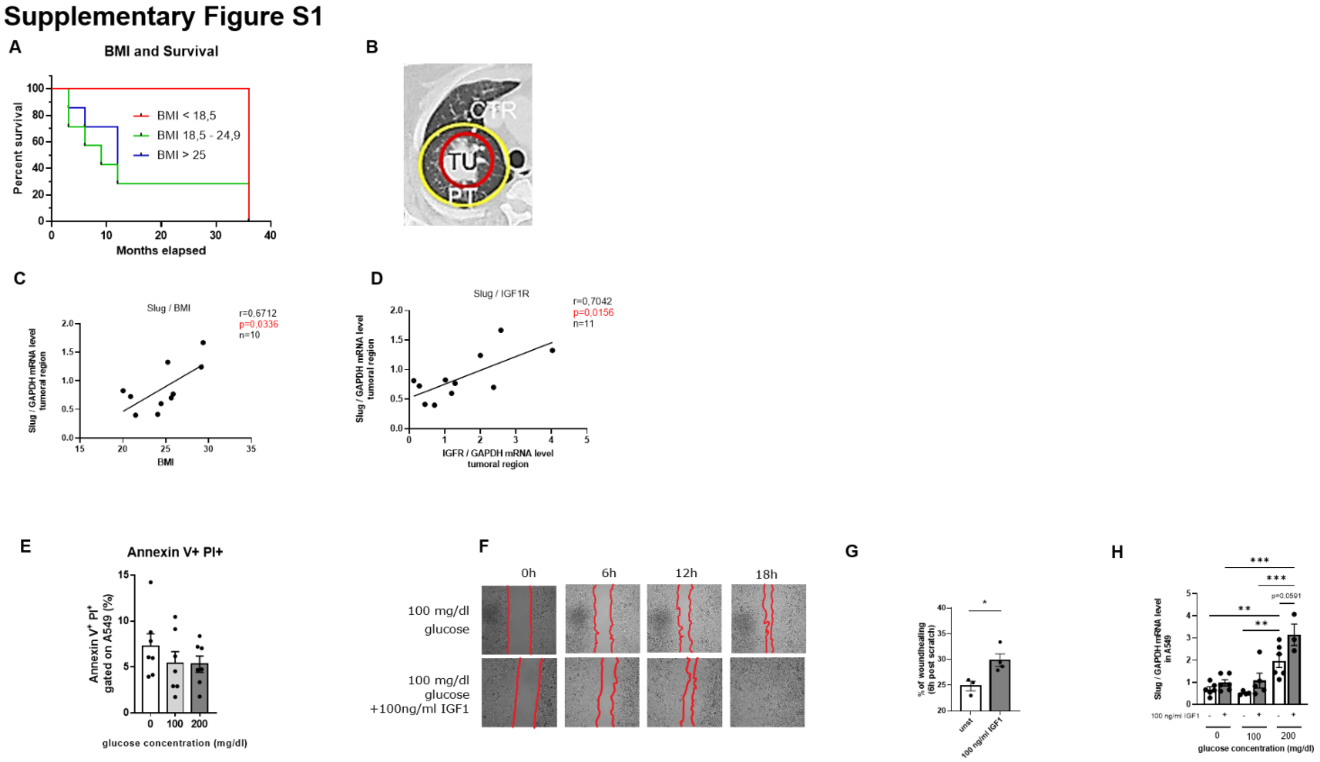

**Supplementary Figure S1. Human cohort of NSCLC patients. A)** Kaplan-Meier curve depending on patients BMI (BMI < 18,5 n=1, BMI 18,5-24,9 n=7 BMI >25 n=7). **B)** CT illustration of the areas for lung tissue resected after surgery of NSCLC patients. **C)** qPCR analysis of relative SLUG/GAPDH mRNA expression level correlated with BMI of patients (n=10), p= 0,0336, r=0,6712, **(B-H** p= 0,05). **D)** qPCR analysis of relative Slug/GAPDH mRNA expression level correlated with relative IGF1R/GAPDH mRNA expression level of patients (n=12); p=0,0156, r= 0,7042 (B-H p= 0,025). **E)** Annexin V/PI FACS analysis of untreated A549 cell culture, cultured in 0, 100 or 200mg/dl glucose; late apoptotic (AnnexinV+ PI+) A549 cells (n=7). **F)** Scratched A549 cell mono layer. Photos were taken 0 h, 6 h, 12 h, 18 h after the scratch to detect cellular migration. Upper panel shows untreated cells with 100 mg/dl glucose in cell culture medium. In the lower panel, A549 cells were treated with 100 ng/ml IGF1 cultured with the same glucose concentration. The scratch is highlighted in red. **G)** Relative migration in percent of scratched A549 cells simulating wound healing capability after 6h; p (unst vs. 100 ng/ml IGF1)=0.0285 (n=4). **H)** qPCR analysis of relative SLUG/GAPDH mRNA expression level in A549 cells cultured as described in figure 3h; p (0 mg/dl glucose without IGF1 vs. 200 mg/dl glucose without IGF1) =0,0063, p (100 mg/dl glucose without IGF1 vs. 200 mg/dl glucose without IGF1)=0,0056, p (100 mg/dl glucose with IGF1 vs. 200 mg/dl glucose with IGF1) =0,0004, p (0 mg/dl glucose with IGF1 vs. 200 mg/dl glucose with IGF1) =0,0001

Correlations are shown using simple linear regression. The two-tailed Pearson correlation analysis was performed to get the r and p value for figures B and C. One-way ANOVA test was used for figure H and unpaired T-Test for G.

## Supplementary Figure S2

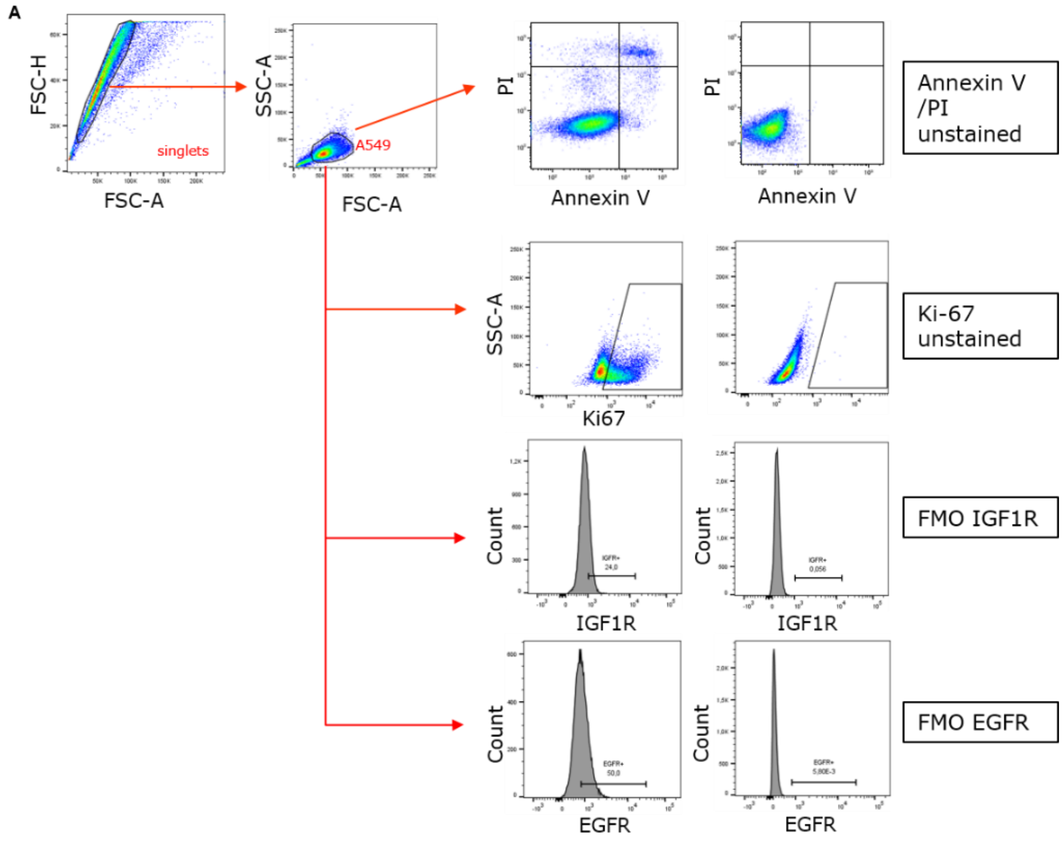


**Supplementary Figure S2. Gating strategy for A549 analysis.**

**A)** Representative flow cytometry staining of A549 sample. For cell preparation, cells were collected and washed. Next, cells were stained with fluorochrome labeled antibodies for the identification of the different expression levels of proteins. Cells that are shown were gated to identify the Ki67, AnnexinV, Pi, IGF1R and EGFR. Therefore, doublets were excluded gating FSC-A against FSC-H. Afterwards, the A549 cells were gated in FSC-A against SSC-A. Next, different expression levels were identified gating SSC-A against the protein of interest or Annexin V against Pi to differentiate early and late apoptosis as well as necrotic cells. For identification of EGFR and IGFR1 plots are shown as histograms and respective FMOs are shown on the right.

**Supplementary Figure S3**

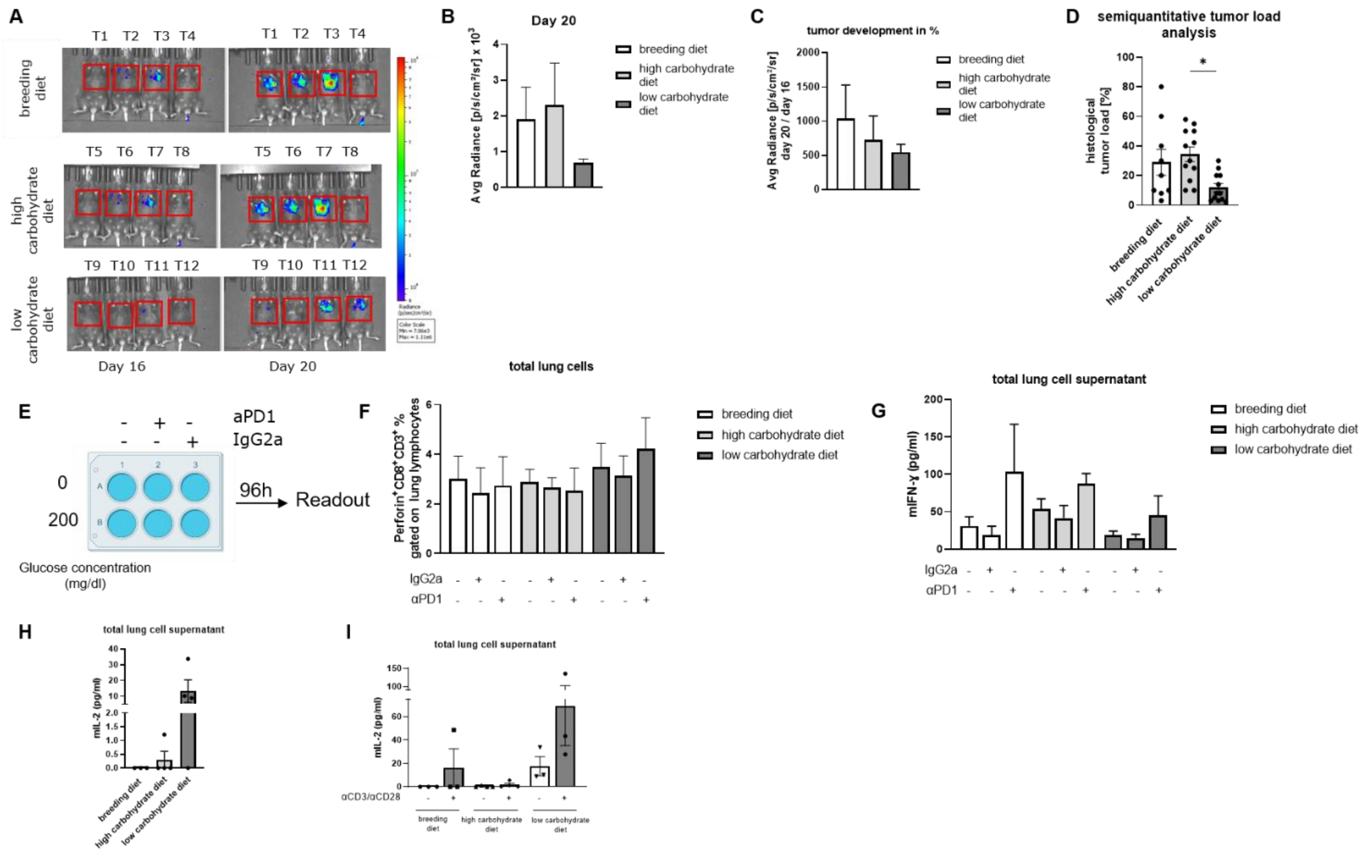


**Supplementary Figure S3. Carbohydrate deprivation diet in a murine model of lung cancer. A)** Representative IVIS pictures from one out of three experiments are shown on day 16 and day 20 with the region of interest (ROI). **B)** Quantitative analysis of in vivo bioluminescence imaging results on day 20. Data are shown as average radiance (p / s / cm2 / sr) of an area 3,17 cm x 33,17 cm. **C)** Tumor development (%) was calculated by the ratio of the average radiance (p / s / cm2 / sr) on day 20 and day 16. **D)** Semiquantitative analysis of total tumor load shown in histology of three independent experiments, analyzed in a blinded study of a pathologist from the Department of Pathology of the University hospital Erlangen ($n^{breeding\ diet}$=9, $n^{high\ carbohydrate\ diet}$=12, $n^{low\ carbohydrate\ diet}$=12), p (high carbohydrate vs. low carbohydrate diet) =0,0128. **E)** Experimental design of isolated murine total lung cells culture for 96h with 0mg/dl glucose or 200mg/dl glucose, depending on mice´s diet. Cells from mice fed with carbohydrate containing diet were cultured with 200 mg/dl glucose in the cell culture medium. Cells from mice fed with the low carbohydrate diet were cultured with 0 mg/dl glucose in the cell culture medium. Additionally, cells were either left unstimulated, treated with αIgG2a or αPD1. **F)** CD3+ CD8+ Perforin+% T-cells of total lung lymphocytes after 5 days cell culture as described above, treated with anti-PD-1 antibody or anti-IgG2a. Cells were collected, stained with antibodies and analyzed by flow cytometry. **G)** Murine IFN-γ concentration of the total lung cell culture supernatant after 5 days either stimulated with αCD3/αCD28, treated with anti-PD-1 antibody or treated with anti-IgG2a antibody and analyzed by ELISA. **H)** Murine IL-2 concentration in total lung cell culture supernatant after 5 days cell culture, measured by ELISA; ($n^{breeding\ diet}$=3, $n^{high\ carbohydrate\ diet}$=3, $n^{low\ carbohydrate\ diet}$=5). **I)** Murine IL-2 concentration in total lung cell culture supernatant after 5 days cell culture and stimulation von αCD3/αCD28 antibodies, measured by ELISA; ($n^{breeding\ diet}$=3, $n^{high\ carbohydrate\ diet}$=4, $n^{low\ carbohydrate\ diet}$=3). One-way ANOVA test was used for figure D.

## Supplementary Figure S4

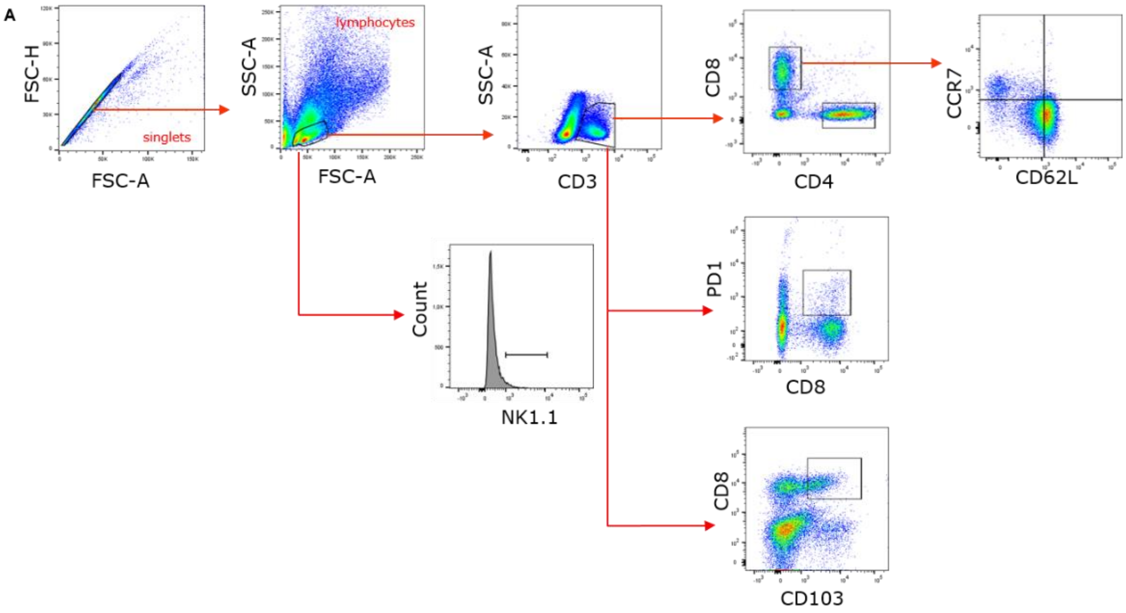


**Supplementary Figure S4. Gating strategy for different lymphocyte subpopulations in murine lung and lymph node. A)** Representative flow cytometry staining of a lung sample from a naïve C57BL/6 mouse with tumor. For lung preparation, the tissue was digested with DNase I/Collagenase D for the isolation of lung single cell suspension. Next, cell suspension was filtered and treated with ACK lysis buffer. Total lung cells were stained with fluorochrome labeled antibodies for the identification of the different lymphocytes subsets. Cells that are shown were gated to identify the TCM, TEM, TRM ($CD3^+CD8^+CCR7^{high}CD62L^{high}$ for TCM), ($CD3^+CD8^+CCR7^{low}CD62L^{low}$ for TEM) for TCM) and ($CD3^+CD8^+CD103^+$ for TRM). Therefore, doublets were excluded gating FSC-A against FSC-H. Afterwards, the lymphocytes are gated in FSC-A against SSC-A. Next, T cells were identified gating on $CD3^+$ cells. Then, $CD4^+$ and $CD8^+$ T cells were identified gating on CD4 and CD8. To identify TCM T cells, we next gated in $CCR7^{high}$ and $CD62L^{high}$. For TEM we gated on $CCR7^{low}$ and $CD62L^{low}$. To identify PD1 positive CD8 T cells we gated from $CD3^+$ on $CD8^+$ and $PD1^+$ cells. We also gated for TRM T cells from $CD3^+$ on $CD8^+$ and $CD103^+$ cells. Finally, data were further analyzed and evaluated with the program FlowJo.
